# Supplementary material for: Cancer Incidence and Mortality among Petroleum Industry Workers and Residents Living in Oil Producing Communities: A Systematic Review and Meta-Analysis
Source: Int J Environ Res Public Health. 2021 Apr 20;18(8):4343. doi: 10.3390/ijerph18084343 (PMC8073871; doi:10.3390/ijerph18084343)
Supplement: Supplementary file 1 [file ijerph-18-04343-s001.zip › ijerph-1157656-supplementary.pdf]

Supplemental Materials

Cancer incidence and mortality among petroleum industry workers and residents living in oil producing communities: A systematic review and meta-analysis

Felix M. Onyije, Bayan Hosseini, Kayo Togawa, Joachim Schüz, and Ann Olsson
Environment and Lifestyle Epidemiology Branch, International Agency for Research on Cancer (IARC/WHO), 150 Cours Albert Thomas, 69372 Lyon CEDEX 08, France
OnyijeF@fellows.iarc.fr

Search Strategy

PubMed: #**1**"Refinery worker*" OR "Petroleum worker*" OR "Oil and gas worker*" OR "Refinery staff" OR "Refinery technic*" OR "Refinery employ*" OR "Petroleum staff" OR "Petroleum employ*" OR "Petroleum technic*" OR "Oil and gas staff*" OR" Oil and gas employ*" OR" Oil and gas technic*" OR "Petroleum"[Mesh] OR Petroleum OR "Petroleum Pollution"[Mesh] OR "Oil and Gas Indust*"[Mesh] OR "Oil and Gas" OR "Oil and Gas Indust*" OR "Crude Oil Indust*" OR "Petroleum refiner*" OR "Oil refiner*" #**2** "Environmental Pollution"[Mesh] OR "Environmental Exposure" OR "Occupational Exposure"[Mesh] #**3** "Neoplasms"[Mesh] OR cancer OR cancers OR cancerous OR carcinoma* OR neoplasm* OR tumor OR tumors OR tumour* OR malignan* OR Leukemia* OR Leukaemia* OR Mesothelioma* OR Myeloma* #**4** "Case-Control Stud*"[Mesh] OR "Case-Control Stud*" OR cohort OR "Cross-Sectional "[Mesh] OR "Cross-Sectional" OR "Epidemiology"[SH] #**5** #1 AND #2 AND #3 AND #4 #**6** "Animals"[MH] NOT Humans [MH] #**7** #5 NOT #6"

Web of Science: #1 TS= ("Refinery worker*" OR "Petroleum worker*" OR "Oil and gas worker*" OR "Refinery staff" OR "Refinery technic*" OR "Refinery employ*" OR "Petroleum staff" OR "Petroleum employ*" OR "Petroleum technic*" OR "Oil and gas staff*" OR" Oil and gas employ*" OR" Oil and gas technic*" OR Petroleum OR "Petroleum Pollution" OR "Oil and Gas" OR "Oil and Gas Indust*" OR "Crude Oil Indust*" OR "Petroleum refiner*" OR "Oil refiner*") #2 TS = ("Environmental Pollution" OR "Environmental Exposure" OR "Occupational Exposure") #3 TS= (Neoplasm* OR cancer OR cancers OR cancerous OR carcinoma* OR tumor OR tumors OR tumour* OR malignan* OR Leukemia* OR Leukaemia* OR Mesothelioma* OR Myeloma*) #4 TS= ("Case-Control Stud*" OR cohort OR "Cross-Sectional" OR Epidemiology) #5 #1 AND #2 AND #3 AND #4"

Table S1- S5: JBI quality appraisal checklist for studies evaluating cancer incidence and mortality among petroleum industry workers and residents of oil producing communities.

Table S1: JBI quality appraisal checklist for cohort studies on petroleum workers

**First author, year**	Free of selection bias?	Clear exposure measurement?	Valid & reliable exposure measurement?	Identified confounders?	Confounder management strategies stated?	Free of outcome sample?	Valid & reliable outcome measure?	Sufficient follow-up time?	Follow-up complete?	Incomplete follow-up strategies?	Appropriate statistical analysis?	Overall
Christie, 1991	Yes	Yes	Yes	Yes	Yes	Yes	Yes	No	Yes	Yes	Yes	10/11
Gun, 2004	Yes	Yes	Yes	Yes	Yes	Yes	Yes	No	Yes	Yes	Yes	10/11
Gun, 2006a	Yes	Yes	Yes	Yes	No	Yes	Yes	Yes	Unclear	Unclear	Yes	8/11
Gun, 2006b	Yes	Yes	Yes	Yes	Yes	Yes	Yes	Yes	Yes	Yes	Yes	11/11
Schnatter, 1993	Yes	Yes	Yes	Unclear	No	Yes	Yes	Yes	Unclear	Yes	Yes	8/11
Lewis, 2003	Yes	Yes	Yes	No	No	Yes	Yes	Yes	Unclear	No	Yes	7/11
Schnatter, 2012	Yes	Yes	Yes	Yes	No	Yes	Yes	Yes	Yes	Yes	Yes	10/11
Schnatter, 2019	Yes	Yes	Yes	Yes	No	Yes	Yes	Yes	Yes	Yes	Yes	10/11
Gennaro, 1994	Yes	Yes	Yes	No	No	Yes	Yes	Yes	No	No	Yes	7/11
Consonni, 1999	Yes	Yes	Yes	Yes	No	Yes	Yes	Yes	Yes	No	Yes	8/11
Pasetto, 2012	Yes	Yes	Yes	Yes	Unclear	Yes	Yes	Yes	Yes	No	Yes	9/11
Bonzini, 2019	Yes	Yes	Yes	No	No	Yes	Yes	Yes	Unclear	No	Yes	7/11
Koh, 2011	Yes	Yes	Yes	No	No	Yes	Yes	No	Unclear	No	Yes	6/11
Koh, 2014	Yes	Unclear	Unclear	No	No	Yes	Yes	No	Yes	Unclear	Yes	5/11
Aas, 2009	Yes	Unclear	Unclear	No	No	Yes	Yes	Yes	Yes	Yes	Yes	7/11
Stenehjem, 2014	Yes	Yes	Yes	No	No	Yes	Yes	No	Yes	Unclear	Yes	7/11
Kirkeleit, 2007	Yes	Yes	Yes	Yes	No	Yes	Yes	Yes	Unclear	Unclear	Yes	8/11
Jarvholm, 1997	Yes	Yes	Yes	No	No	Yes	Yes	Yes	Yes	Unclear	Yes	7/11
Rushton,1993	Yes	Yes	Yes	No	No	Yes	Yes	Yes	Yes	Unclear	Yes	8/11
Sorahan, 2007	Yes	Yes	Yes	No	No	Yes	Yes	Yes	Yes	Unclear	Yes	8/11
Satin, 1996	Yes	Yes	Yes	No	No	Yes	Yes	Yes	Yes	Unclear	Yes	8/11
Divine,1999a	Yes	Yes	Yes	No	No	Yes	Yes	Yes	Unclear	No	Yes	7/11
Gamble, 2000	Yes	Yes	Yes	Yes	No	Yes	Yes	Yes	Unclear	No	Yes	8/11
Huebner, 2000	Yes	Unclear	Yes	No	No	Yes	Yes	No	Yes	Yes	Yes	8/11
Sathiakumar, 2001	Yes	Yes	Yes	Yes	Yes	Yes	Yes	No	Yes	No	Yes	9/11
Wong, 2001a	Yes	Yes	Yes	Yes	No	Yes	Yes	Yes	Yes	Unclear	Yes	911
Satin, 2002	Yes	Yes	Yes	No	No	Yes	Yes	Yes	Yes	Unclear	Yes	8/11
Tsai 2003	Yes	Yes	Yes	No	No	Yes	Yes	Yes	Yes	No	Yes	8/11
Huebner, 2004	Yes	Yes	Yes	No	No	Yes	Yes	Yes	Yes	Unclear	Yes	8/11
Buffler, 2004	Yes	Yes	Yes	Yes	No	Yes	Yes	Yes	Unclear	Unclear	Yes	8/11
Tsai, 2004	Yes	Yes	Yes	Yes	No	Yes	Yes	No	Unclear	Unclear	Yes	7/11

Tsai, 2007	Yes	Yes	Yes	Yes	No	Yes	Yes	Yes	Unclear	Unclear	Yes	8/11
Huebner 2009	Yes	Yes	Yes	No	No	Yes	Yes	Yes	Yes	Yes	Yes	9/11
Divine, 1999b	Yes	Yes	Yes	No	No	Yes	Yes	Yes	Yes	No	Yes	8/11
Wong, 2001b	Yes	Yes	Yes	Yes	No	Yes	Yes	Yes	Unclear	No	Yes	8/11
**Percentage (%)**	**100**	**91**	**94**	**54**	**11**	**100**	**100**	**77**	**66**	**26**	**100**	**74**

Yes=1, No=0, Unclear =0

Table S2: JBI critical appraisal checklist for case-control studies on petroleum workers

**First author, year [reference]**	Free of selection bias?	Appropriate sampling?	The same criteria used for cases and control?	Standard, valid and reliable exposure measurement?	Exposure measured the same way for cases and control?	Identified confounders?	Confounder management strategies stated?	Valid & reliable outcome measure?	Sufficient exposure time?	Appropriate statistical analysis?	Overall
Finkelstein, 1996	Yes	Yes	Yes	Yes	Unclear	No	No	Yes	Unclear	Yes	6/10
Schnatter,1996	Yes	Yes	Yes	Yes	Yes	Yes	Yes	Yes	Unclear	Yes	9/10
Anttila, 2015	Yes	Yes	Yes	Yes	Yes	Yes	Yes	Yes	Yes	Yes	10/10
Rushton, 2014	Yes	Yes	Yes	Yes	Yes	Yes	Yes	Yes	Unclear	Yes	9/10
Glass, 2014	Yes	Yes	Yes	Yes	Yes	Yes	Yes	Yes	Unclear	Yes	9/10
Stenehjem, 2015	Unclear	No	No	Yes	Yes	Yes	Yes	Yes	Unclear	Yes	6/10
Rushton,1997	Yes	Yes	Yes	Yes	Yes	Yes	Yes	Yes	Unclear	Yes	9/10
Poole, 1993	Yes	Yes	Yes	Yes	Yes	Yes	Yes	Yes	Unclear	Yes	9/10
**Percentage (%)**	**88**	**88**	**88**	**100**	**88**	**88**	**88**	**100**	**13**	**100**	**84**

Yes=1, No=0, Unclear =0

Table S3: JBI critical appraisal checklist for cohort studies on residents living near petroleum facilities

**First author, year [reference]**	Free of selection bias?	Clear exposure measurement?	Valid & reliable exposure measurement?	Identified confounders?	Confounder management strategies stated?	Free of outcome sample?	Valid & reliable outcome measure?	Sufficient follow-up time?	Follow-up complete?	Incomplete follow-up strategies?	Appropriate statistical analysis?	Overall
Hurtig, 2002	Yes	Unclear	No	Yes	No	Unclear	Yes	No	No	No	Yes	4/11
Hurtig, 2004	Yes	Yes	Unclear	No	No	Unclear	Yes	Yes	No	Yes	Yes	6/11
Kelsh, 2009	Yes	Yes	Yes	No	No	Unclear	Yes	No	No	No	Yes	5/11
Ramis, 2012	Unclear	Unclear	Yes	Yes	No	Unclear	Yes	No	No	No	Yes	4/11
Barregard, 2009	Unclear	Unclear	Unclear	No	No	Unclear	Yes	Yes	Yes	Yes	Yes	5/11
Sans, 1995	Unclear	Unclear	Unclear	Yes	Yes	Unclear	Yes	No	No	No	Yes	4/11
**Percentage (%)**	**50**	**33**	**33**	**33**	**17**	**0**	**100**	**33**	**17**	**33**	**100**	**41**

Yes=1, No=0, Unclear =0

Table S4: JBI critical appraisal checklist for cross-sectional studies on residents living near petroleum facilities

First author, year [reference]	Criteria for inclusion clearly defined?	Subjects and setting described in detail?	Valid and reliable exposure measurement?	Objective, standard criteria used for measurement of the condition?	Identified confounders?	Confounder management strategies stated?	Valid & reliable outcome measure?	Appropriate statistical analysis?	Overall
Choi, 2018	Yes	Yes	Yes	Unclear	No	No	Yes	Yes	5/8
Lyons, 1995	Yes	Yes	Yes	Yes	No	No	Yes	Yes	6/8
**Percentage (%)**	**100**	**100**	**100**	**50**	**0**	**0**	**100**	**100**	**61**

Yes=1, No=0, Unclear =0

Table S5: JBI critical appraisal checklist for case-control studies on residents living near petroleum facilities

First author, year [reference]	Free of selection bias?	Appropriate sampling?	The same criteria used for cases and control?	Standard, valid and reliable exposure measurement?	Exposure measured the same way for cases and control?	Identified confounders?	Confounder management strategies stated?	Valid & reliable outcome measure?	Sufficient exposure time?	Appropriate statistical analysis?	Overall
Tsai, 2009	No	No	No	Yes	Yes	Yes	Yes	Yes	Unclear	Yes	6/10
Weng, 2008	Yes	Yes	Yes	Unclear	Yes	Yes	Yes	Yes	Unclear	Yes	8/10
Liu, 2008	Yes	Yes	Yes	Unclear	Yes	Yes	Yes	Yes	Unclear	Yes	8/10
Yu, 2005	Yes	Yes	Yes	Unclear	Yes	No	No	Yes	Unclear	Yes	6/10
Micheli, 2014	Yes	Yes	Yes	Unclear	Yes	Yes	Yes	Yes	Unclear	Yes	8/10
McKenzie, 2017	Unclear	No	Unclear	Unclear	Yes	Yes	Yes	Yes	Yes	Yes	6/10
**Percentage (%)**	**67**	**67**	**67**	**17**	**100**	**83**	**83**	**100**	**17**	**100**	**70**

Yes=1, No=0, Unclear =0

Figure S1: Forest and funnel plot of studies evaluating cancer incidence and mortality among petroleum workers

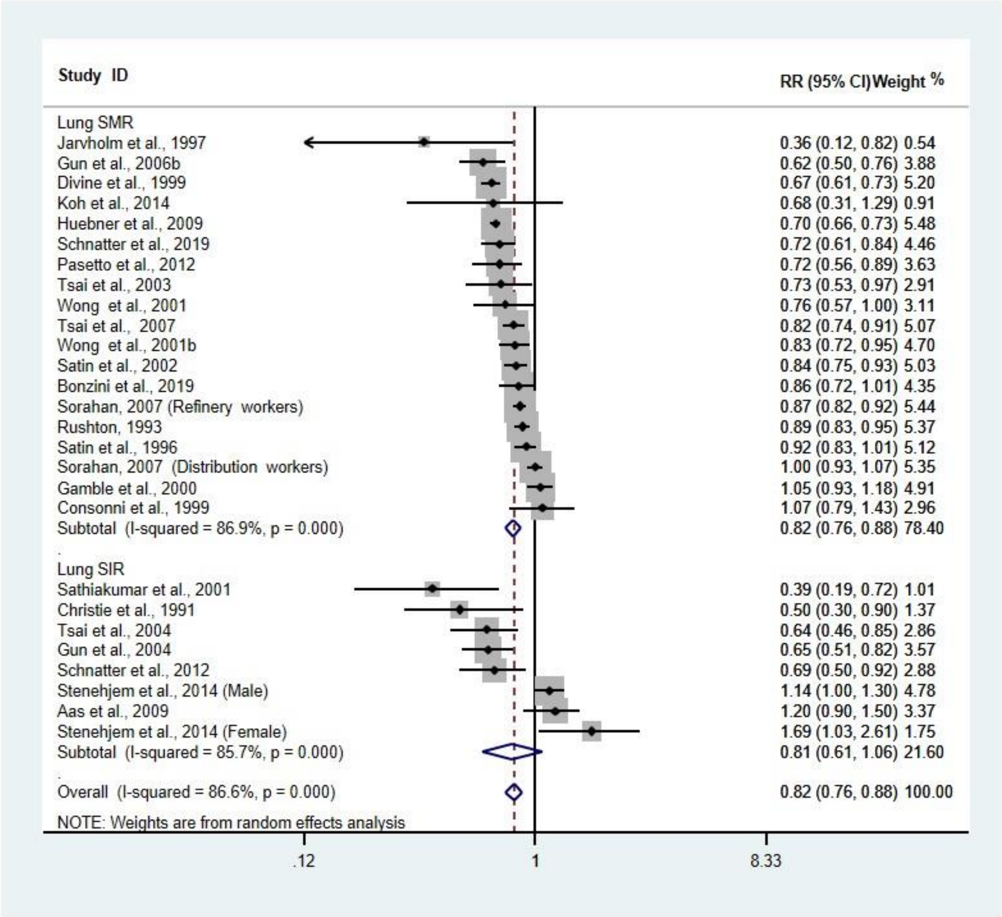

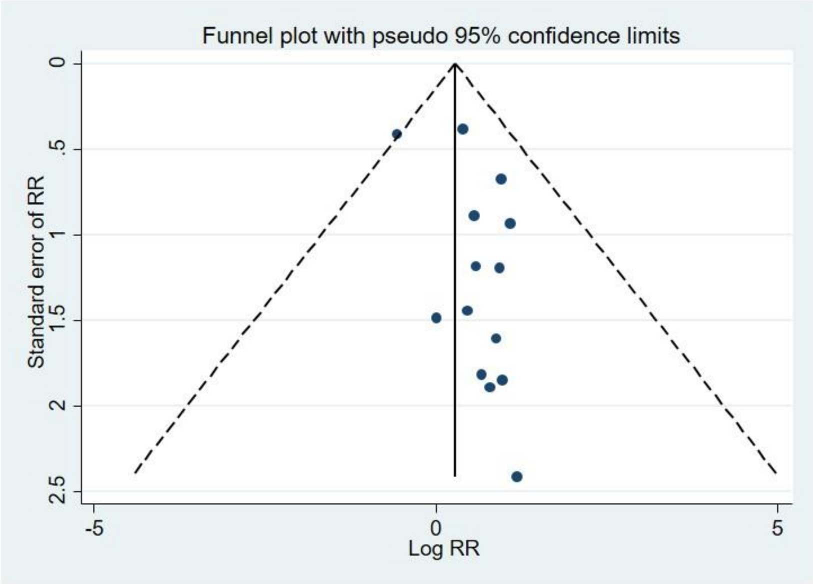

Figure S1a: Forest and funnel plot of studies evaluating lung cancer incidence and mortality among petroleum workers. Egger's regression test for Lung cancer publication bias P = 0.990

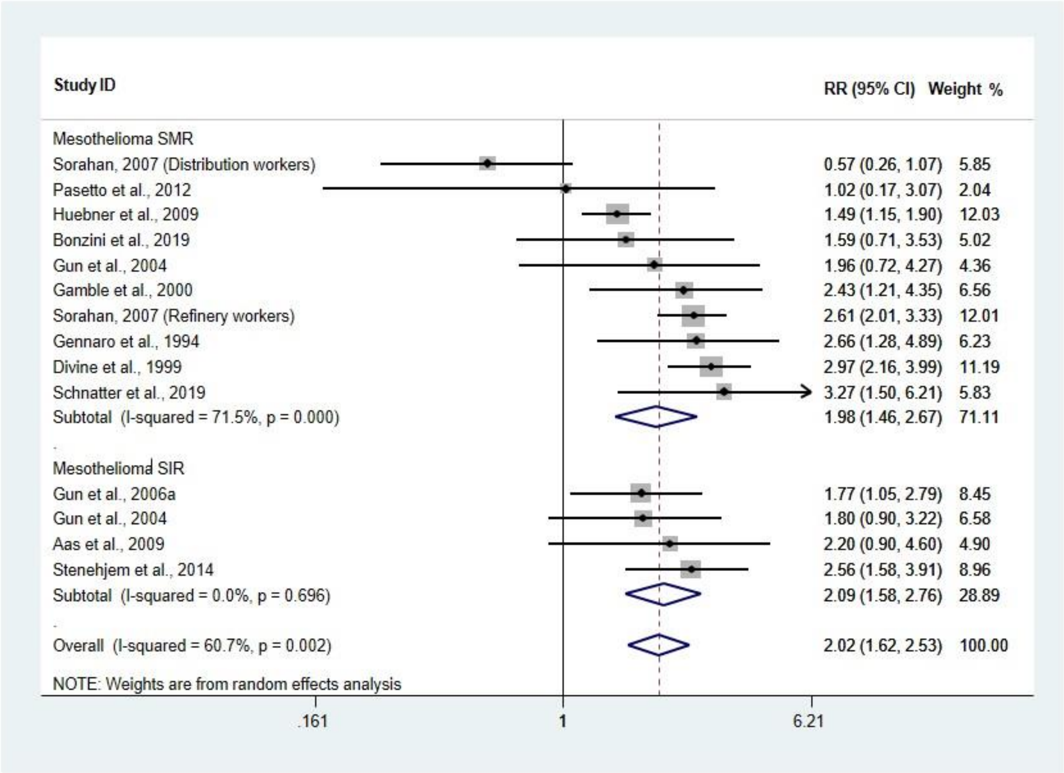

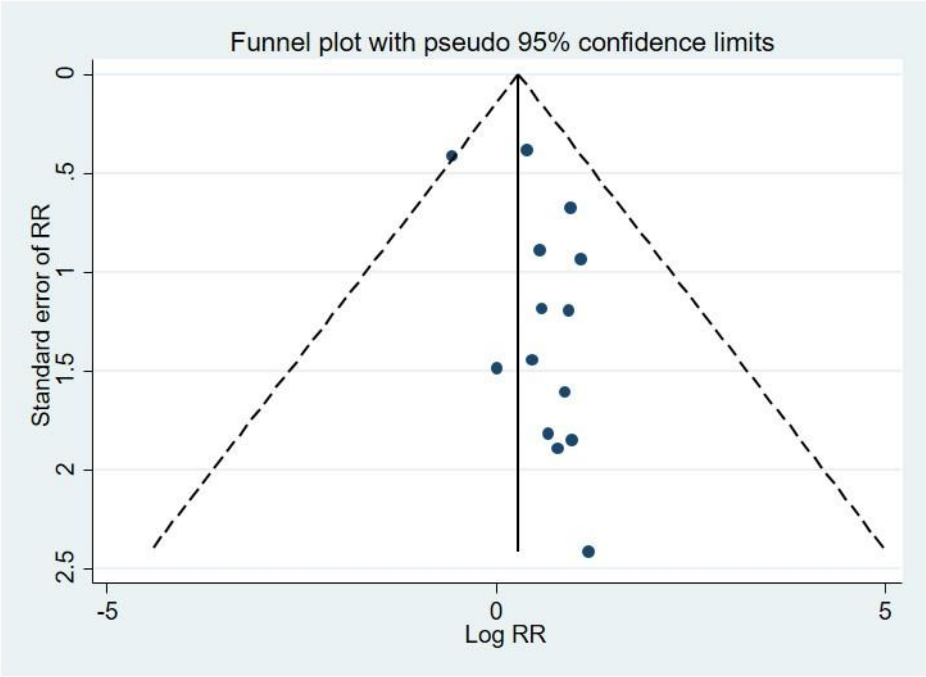

Figure S1b: Forest and funnel plot of studies evaluating mesothelioma incidence and mortality among petroleum workers. Egger's regression test for mesothelioma publication bias P = = 0.066

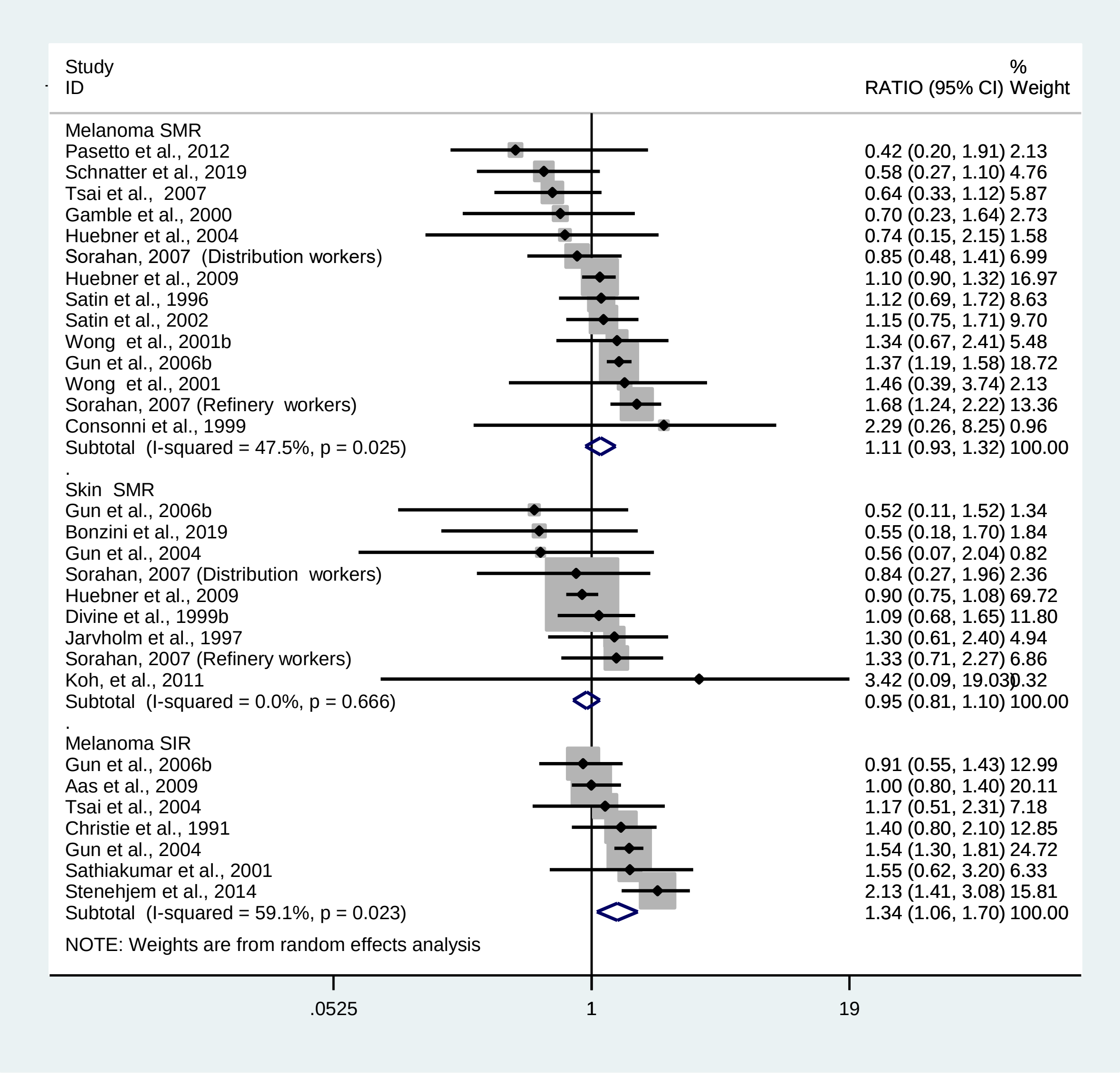

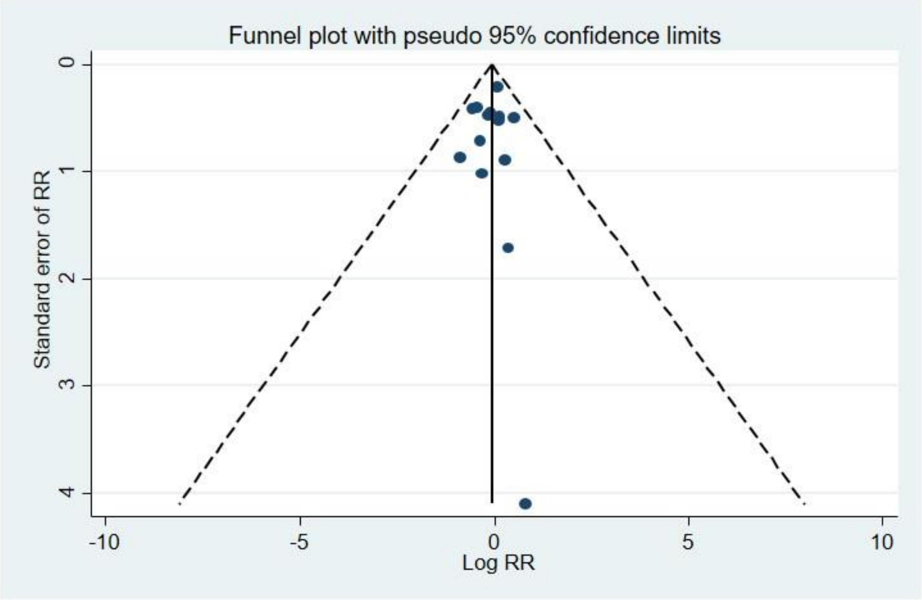

Egger's regression test for melanoma SMR publication bias P = 0.661

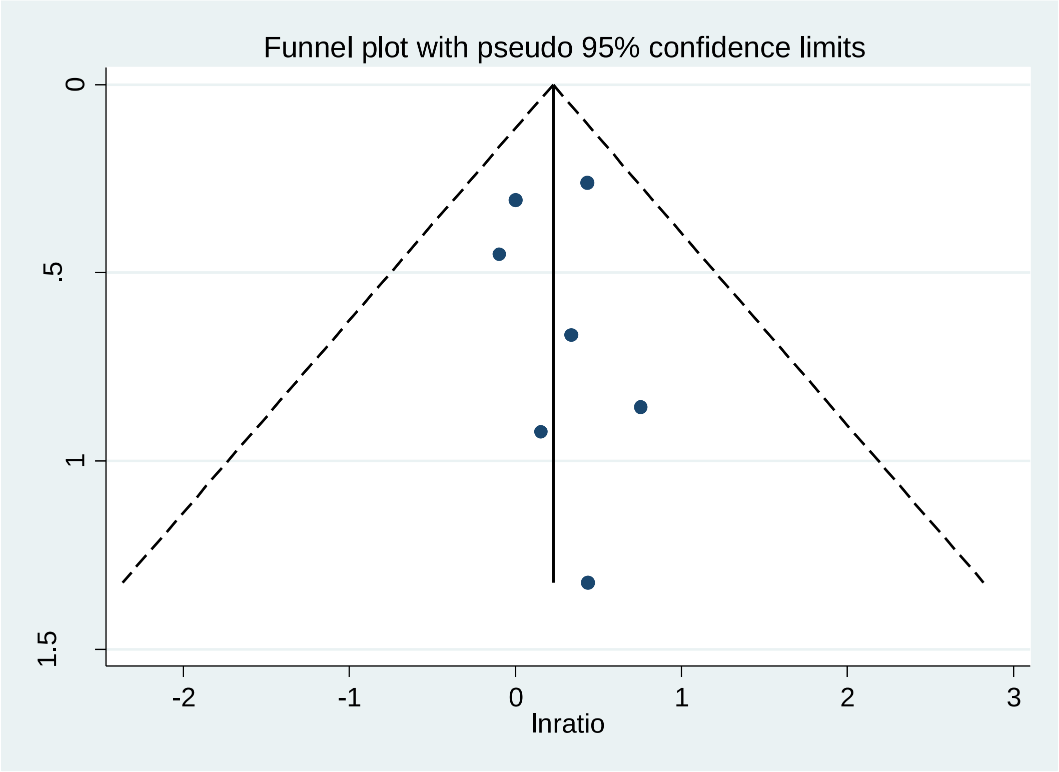

Egger's regression test for melanoma SIR publication bias P = 0.827

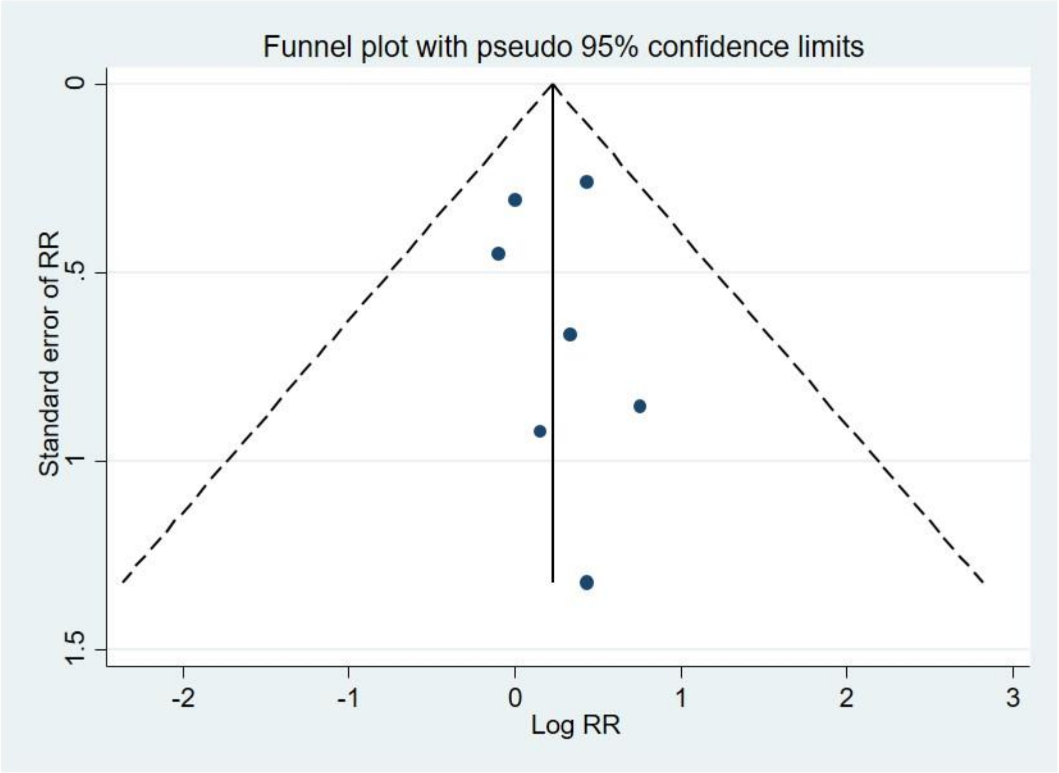

Egger's regression test for skin cancer publication bias P = 0.745

Figure S1c: Forest and funnel plot of studies evaluating melanoma and other skin cancer incidence and mortality among petroleum workers.

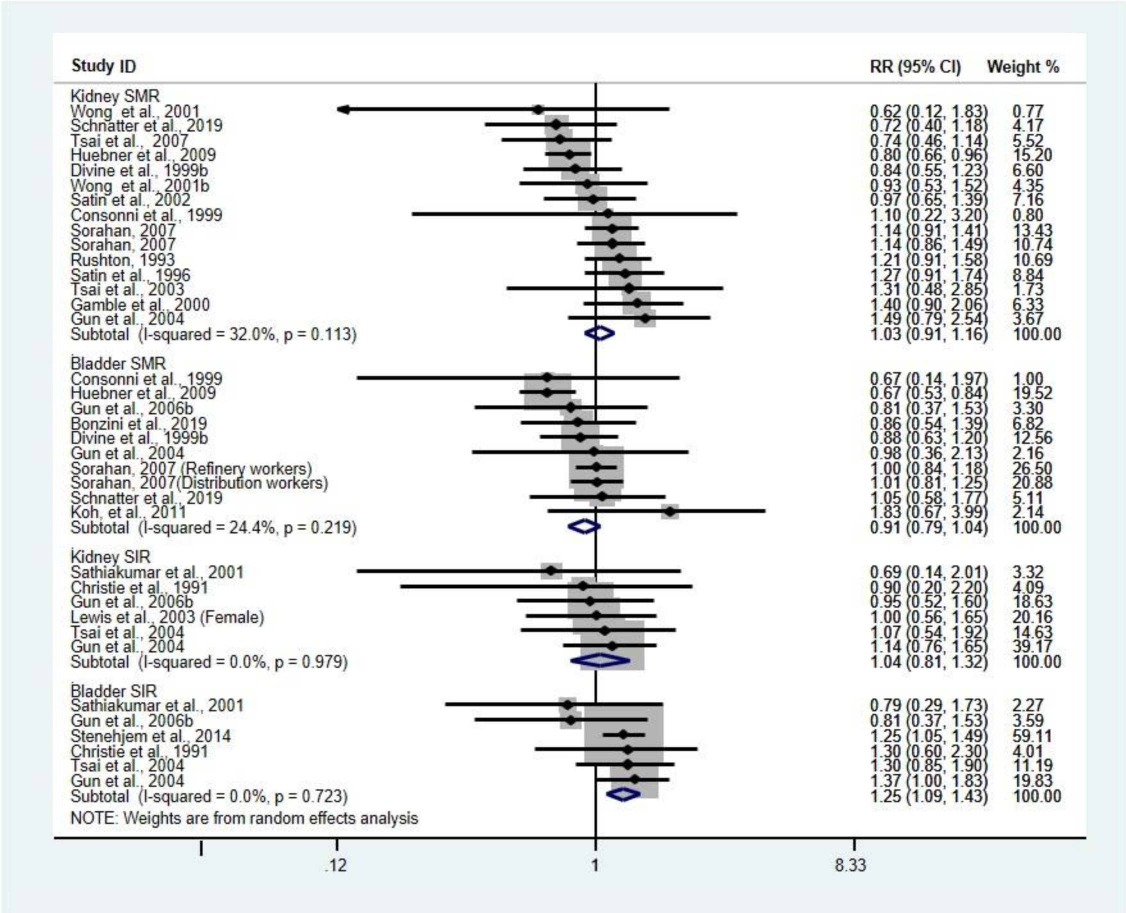

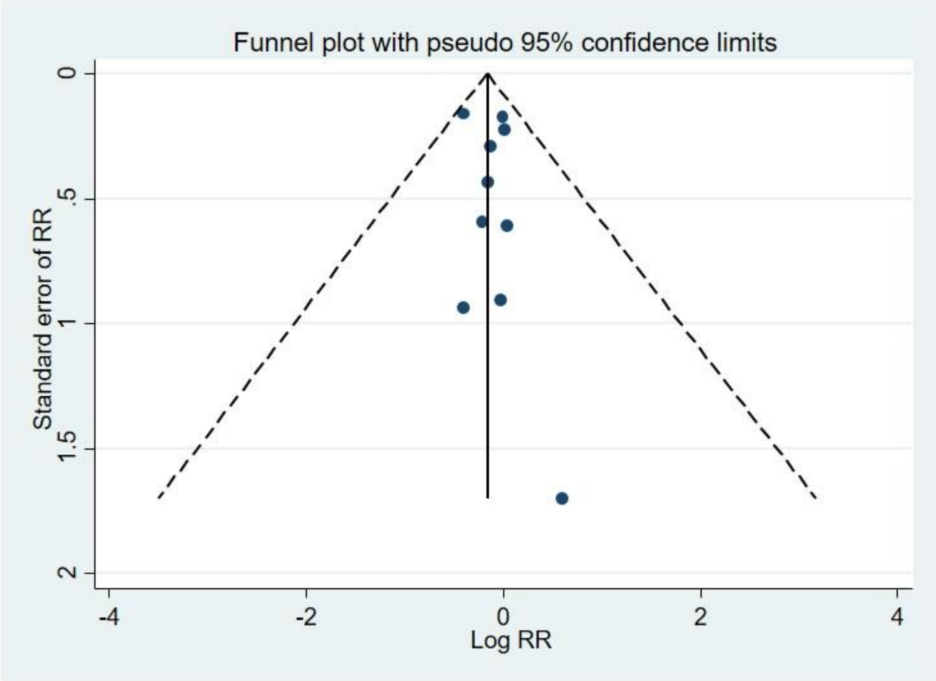

Egger's regression test for *Urinary bladder SMR* publication bias P = 0.504

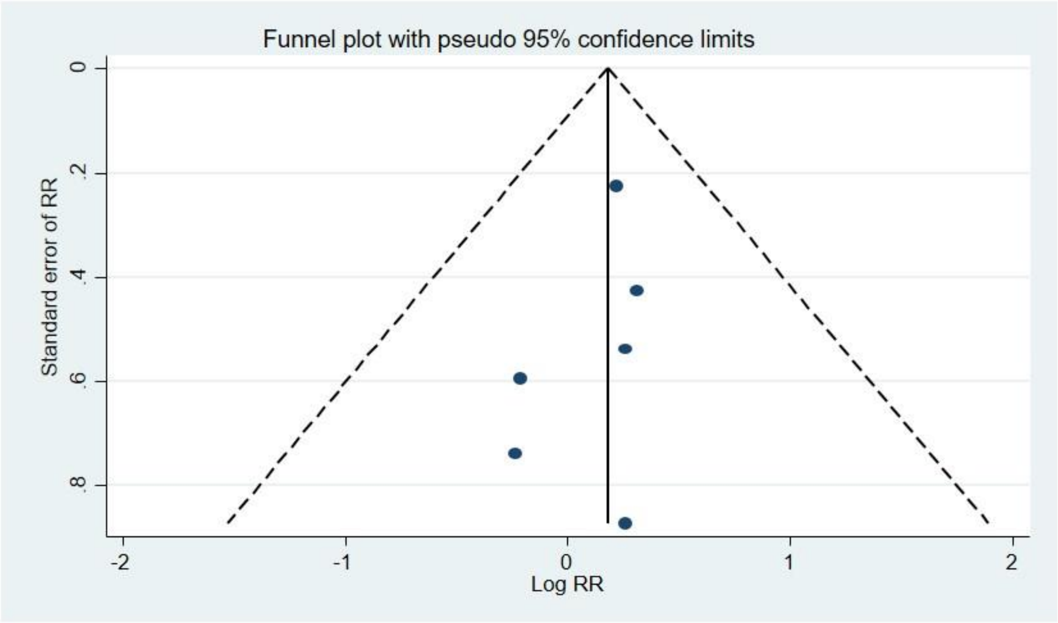

Egger's regression test for *Urinary bladder SIR* publication bias P = 0.341

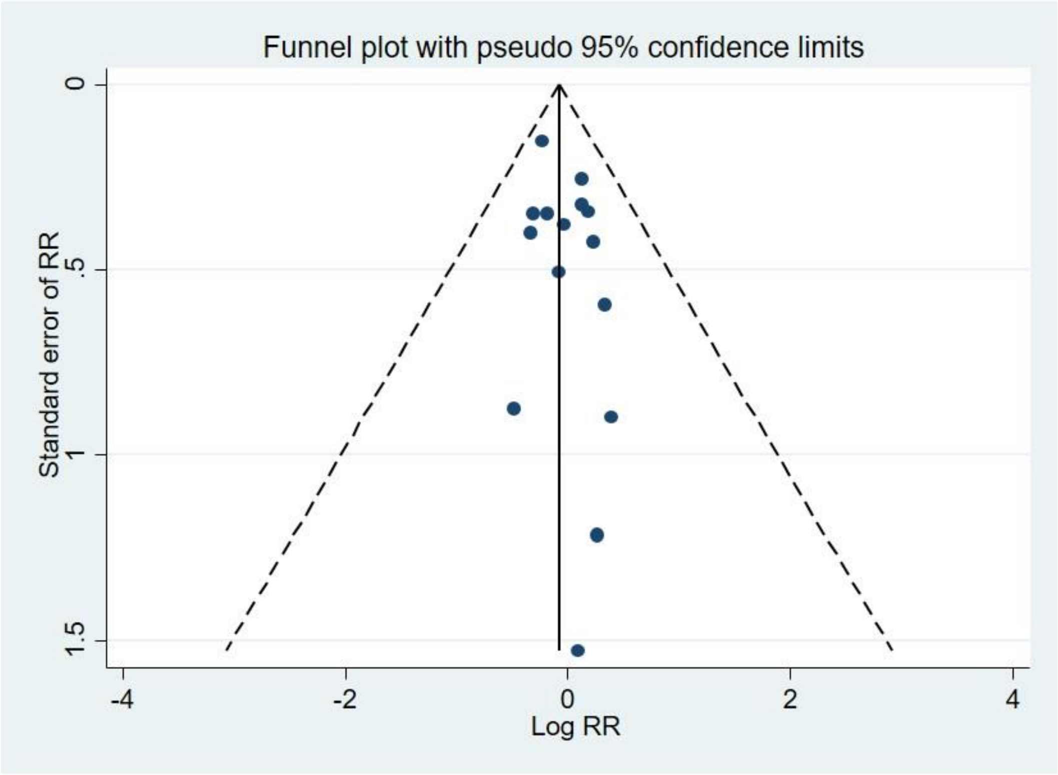

Egger's regression test for *Kidney SMR* publication bias P = 0.184

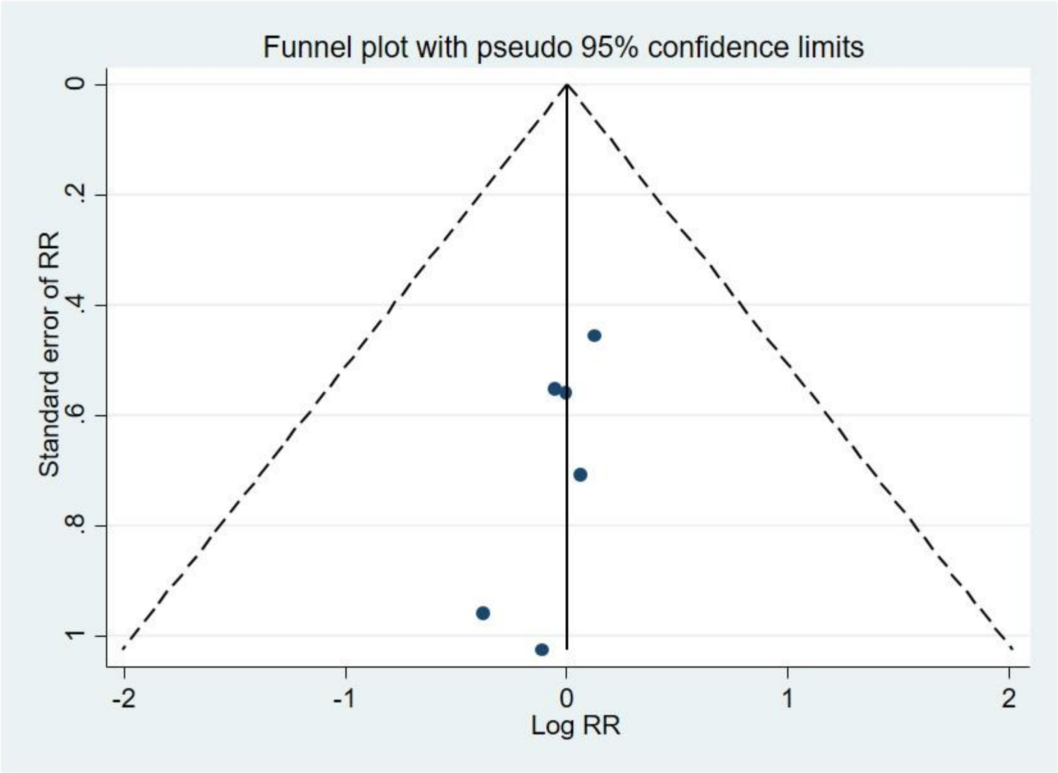

Egger's regression test for *Kidney SIR* publication bias P = 0.085

Figure S1d: Forest and funnel plot of studies evaluating urinary cancer incidence and mortality among petroleum workers.

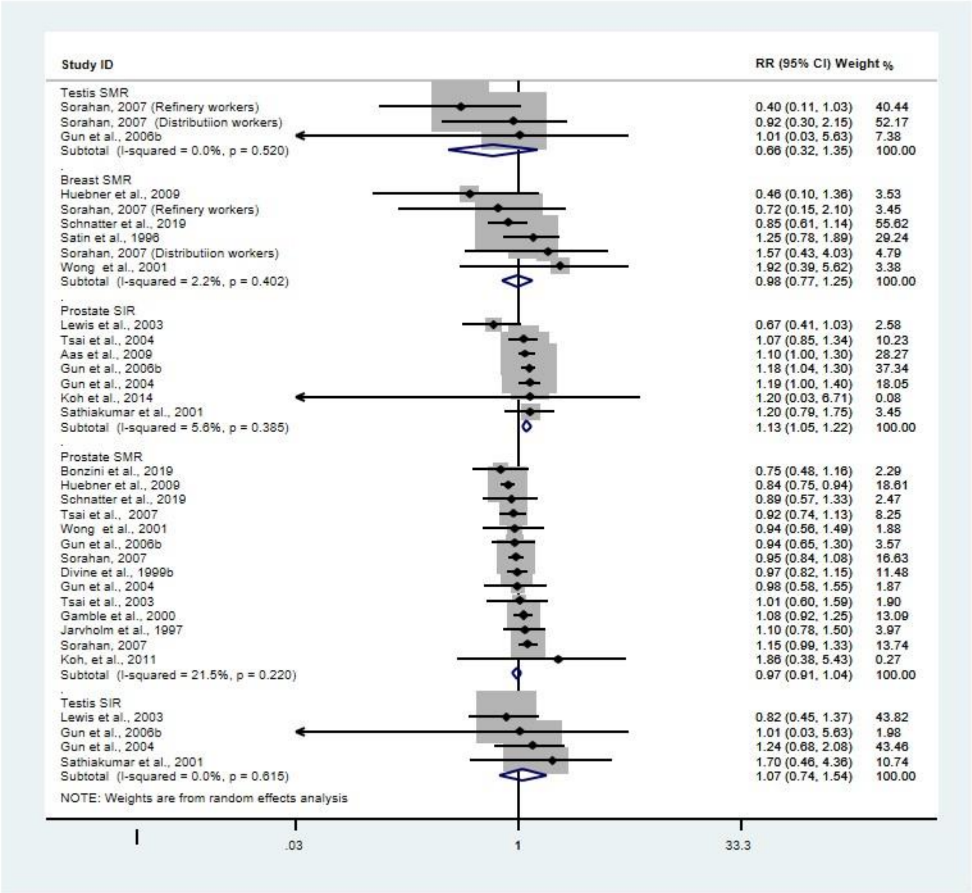

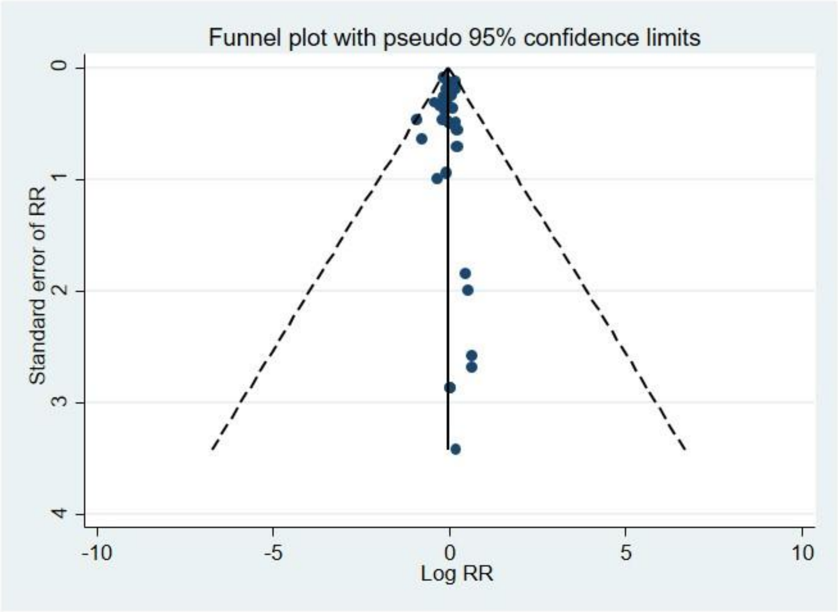

Figure S1e: Forest and funnel plot of studies evaluating reproductive cancer incidence and mortality among petroleum workers**.** Egger's regression test for Reproductive cancer publication bias P = 0.747

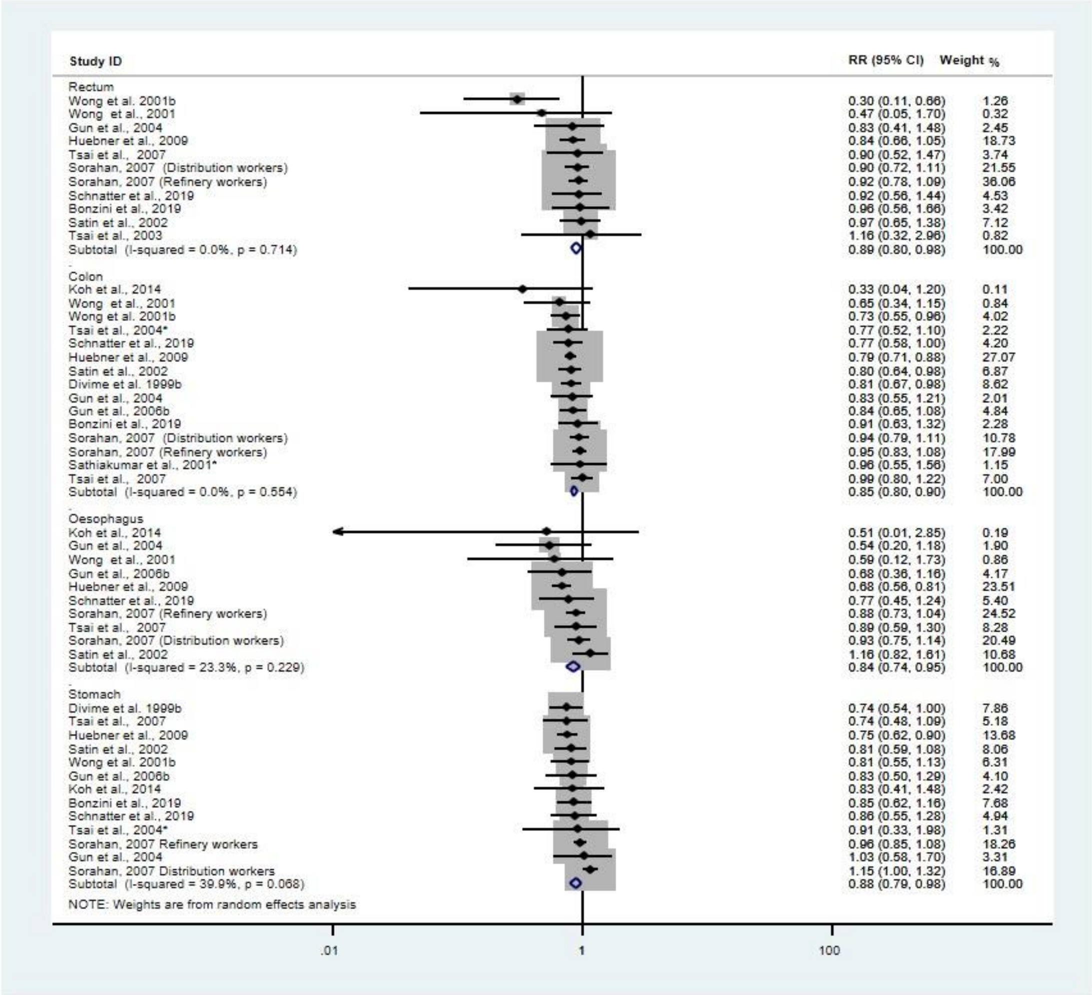

Incidence cases

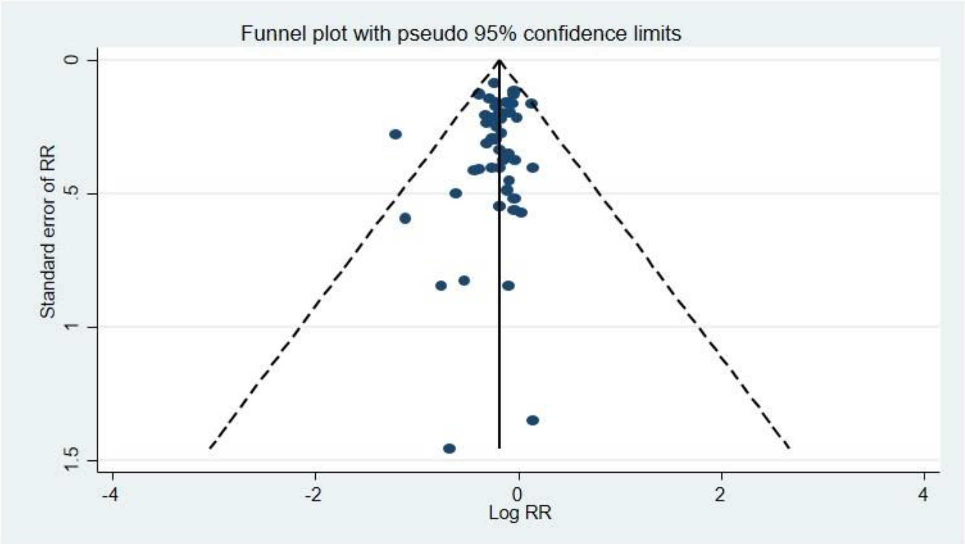

Figure S1f: Forest plot of studies digestive organ cancer incidence and mortality among petroleum workers

Egger's regression test for digestive organ cancer publication bias P = 0.302

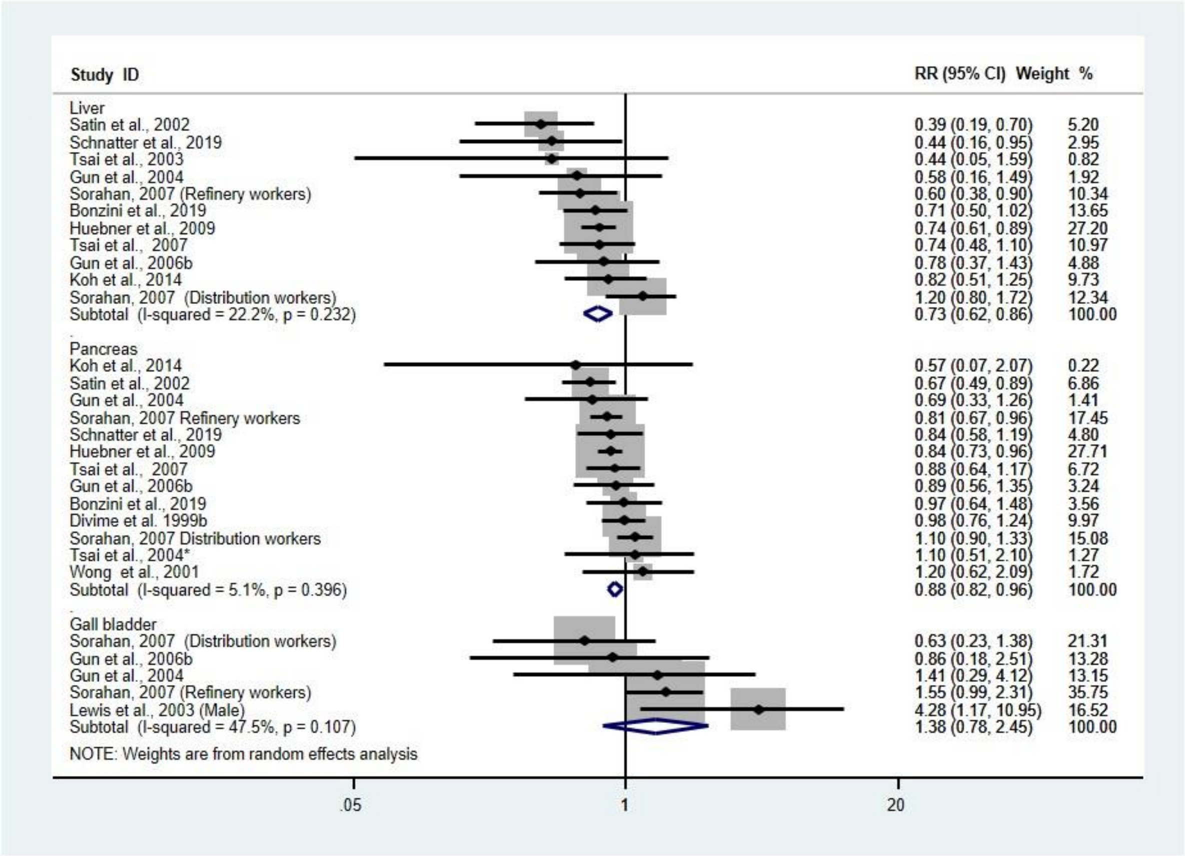

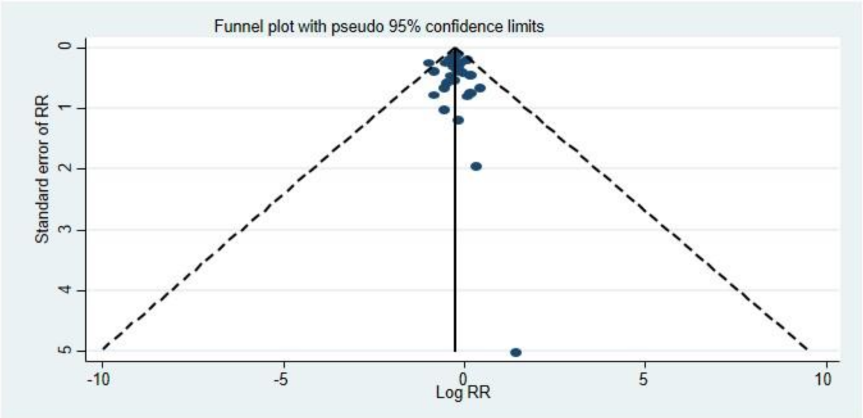

Figure S1g: Forest and funnel plot of studies accessory digestive organ cancer incidence and mortality among petroleum workers. Egger's regression test for Accessory digestive organ cancer incidence and mortality
publication bias P = 0.977

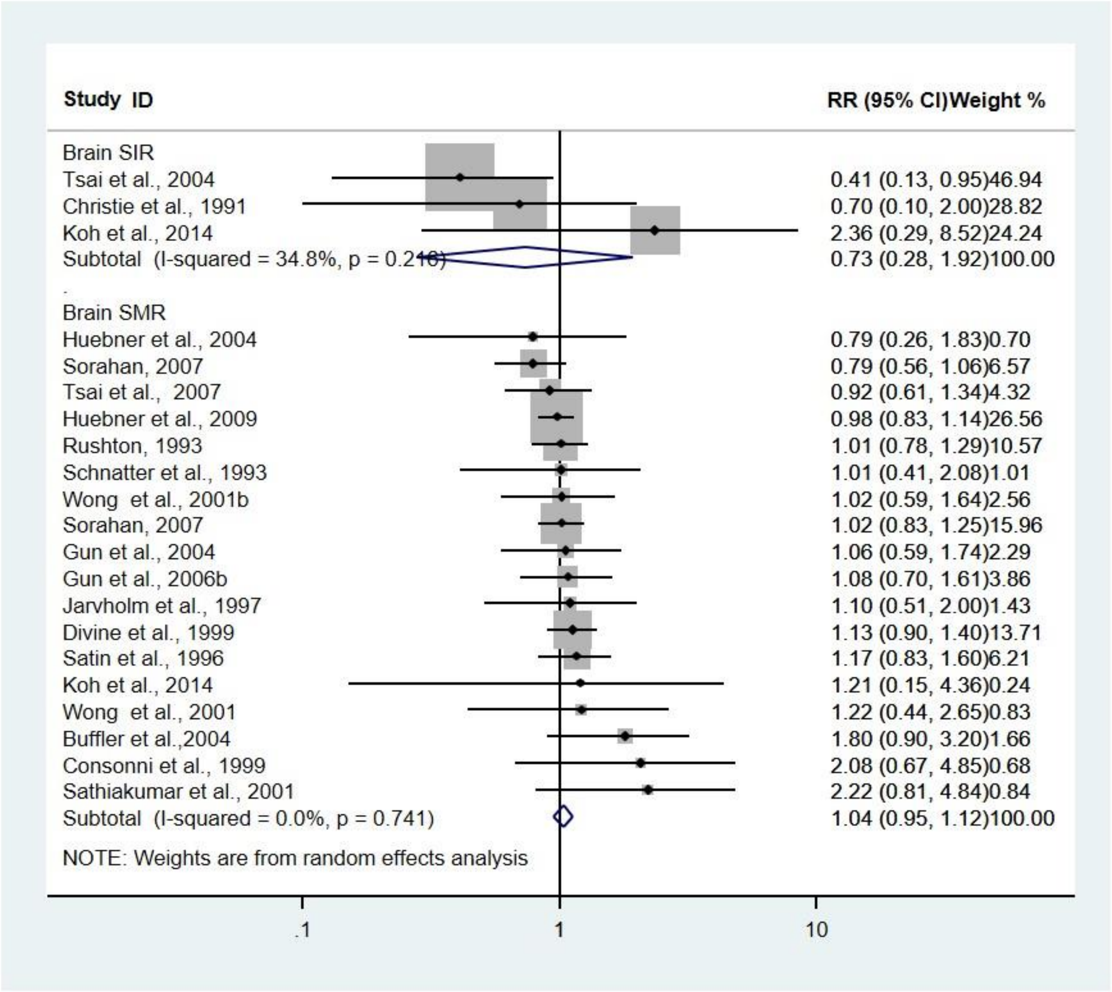

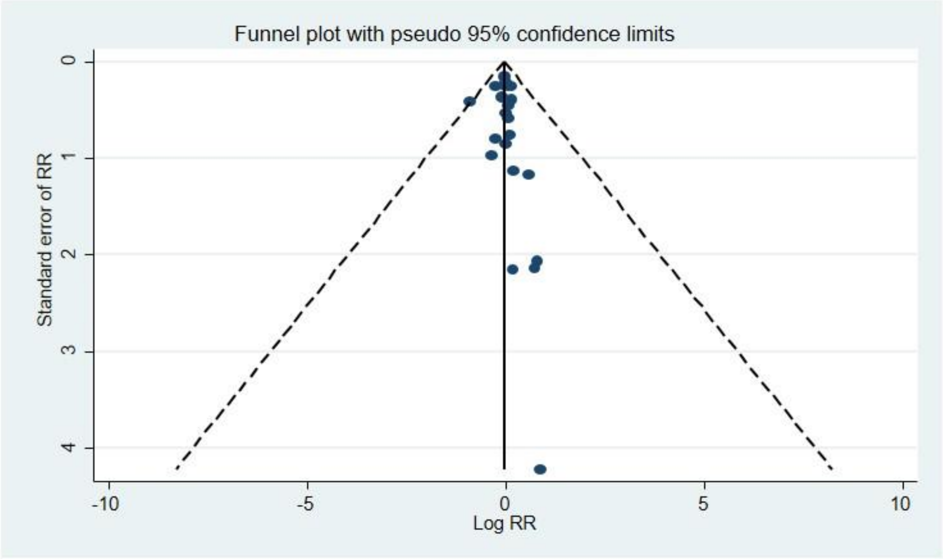

Figure S1h: Forest and funnel plot of studies brain cancer incidence and mortality among petroleum workers

Egger's regression test for brain cancer incidence and mortality publication bias P = 0.632

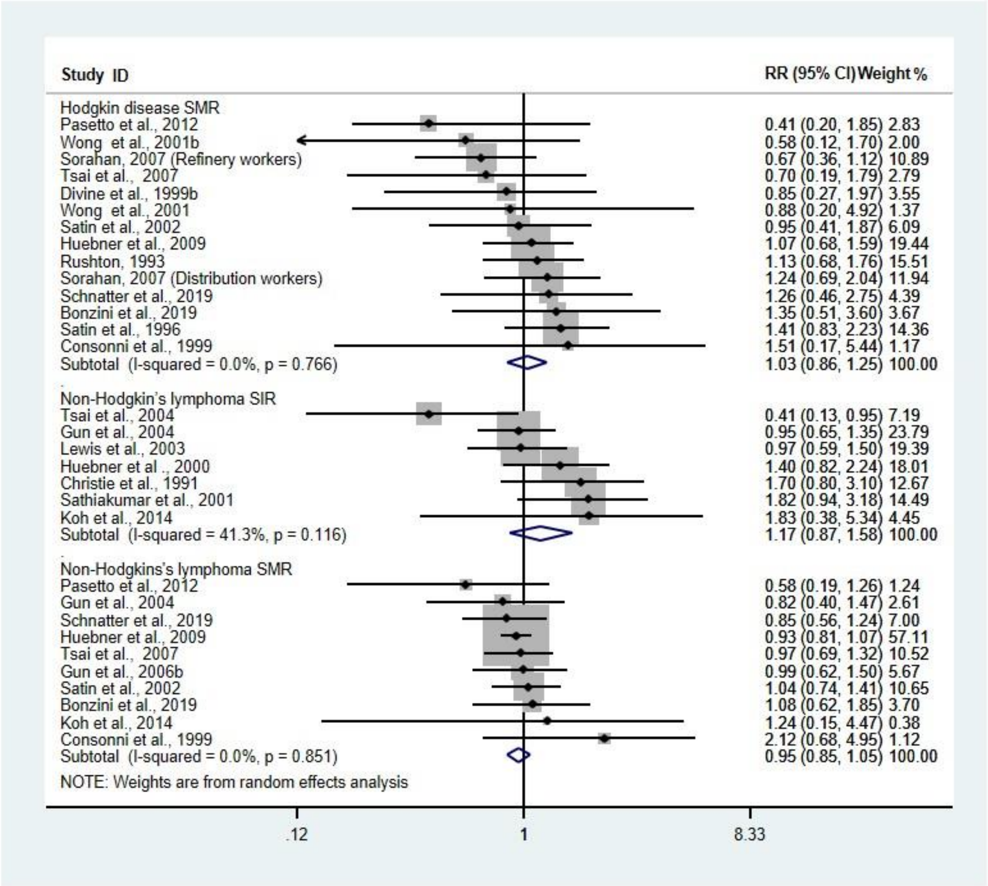

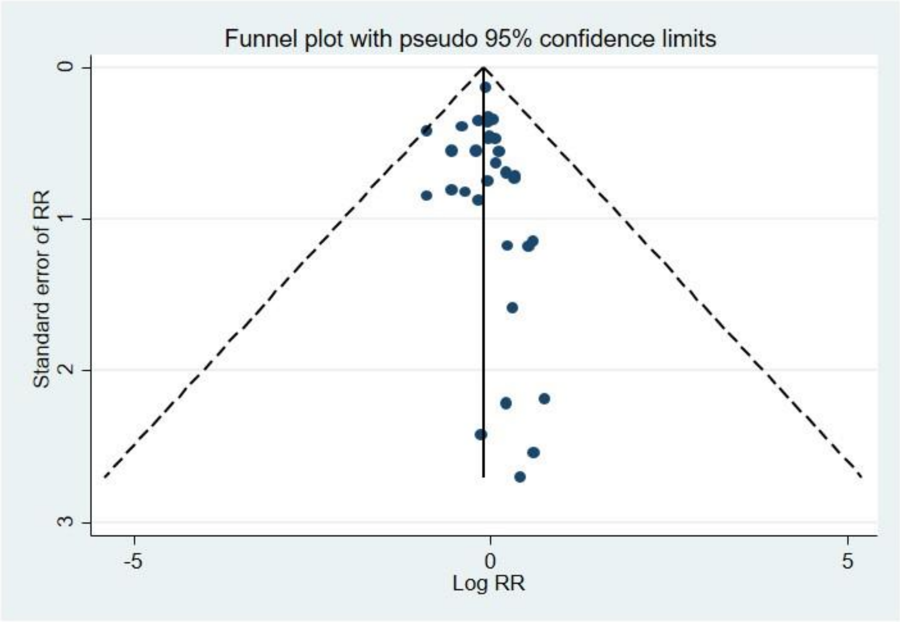

Figure S1i: Forest and funnel plot of studies for Hodgkin's and Non-Hodgkin's Lymphoma incidence and mortality among petroleum workers**.** Egger's regression test HL and NHL incidence and mortality publication bias P = 0.533

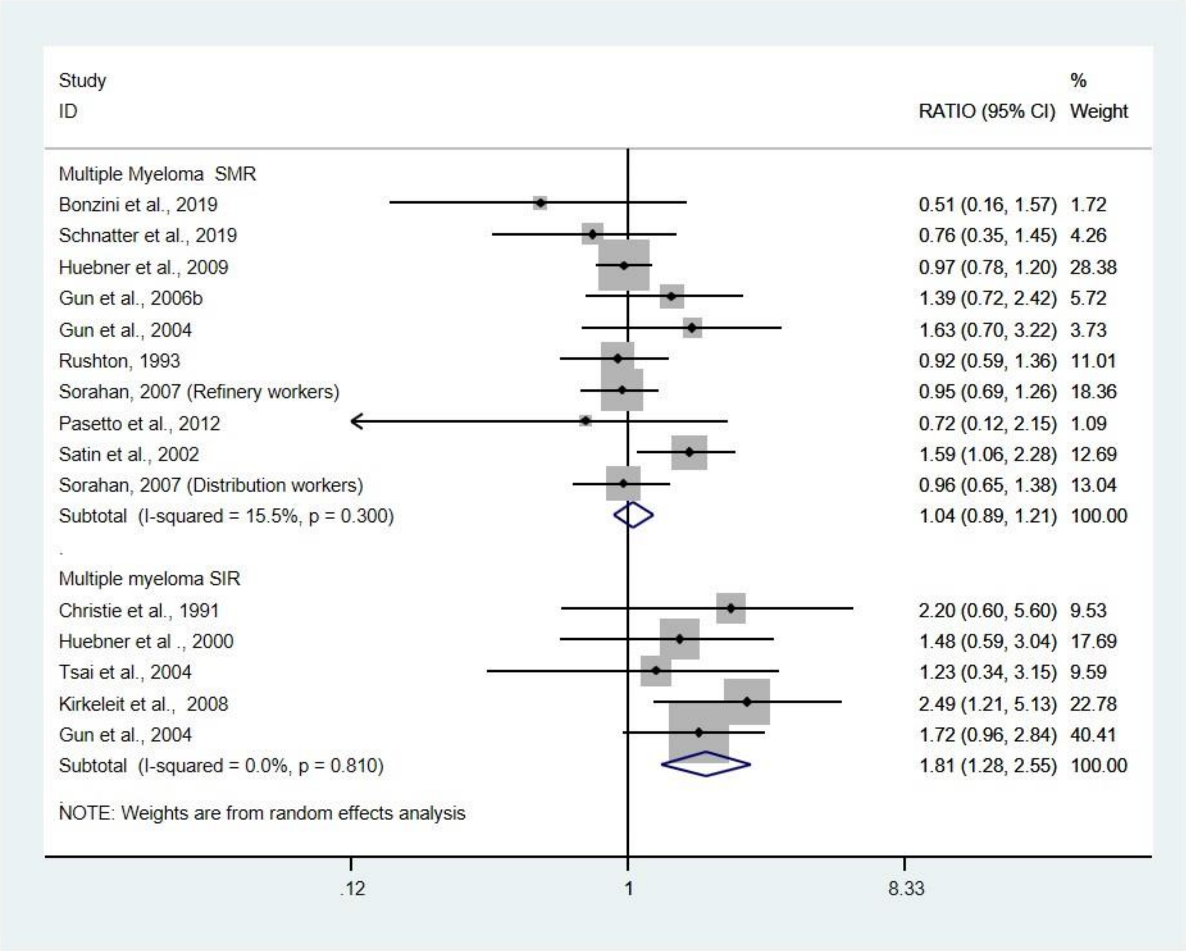

Study
ID
RATIO (95% CI)
%
Weight
Multiple Myeloma SMR
Bonzini et al., 2019 0.51 (0.16, 1.57) 1.72
Schnatter et al., 2019 0.76 (0.35, 1.45) 4.26
Huebner et al., 2009 0.97 (0.78, 1.20) 28.38
Gun et al., 2006b 1.39 (0.72, 2.42) 5.72
Gun et al., 2004 1.63 (0.70, 3.22) 3.73
Rushton, 1993 0.92 (0.59, 1.36) 11.01
Sorahan, 2007 (Refinery workers) 0.95 (0.69, 1.26) 18.36
Pasetto et al., 2012 0.72 (0.12, 2.15) 1.09
Satin et al., 2002 1.59 (1.06, 2.28) 12.69
Sorahan, 2007 (Distribution workers) 0.96 (0.65, 1.38) 13.04
Subtotal (I-squared = 15.5%, p = 0.300) 1.04 (0.89, 1.21) 100.00
Multiple myeloma SIR
Christie et al., 1991 2.20 (0.60, 5.60) 9.53
Huebner et al ., 2000 1.48 (0.59, 3.04) 17.69
Tsai et al., 2004 1.23 (0.34, 3.15) 9.59
Kirkeleit et al., 2008 2.49 (1.21, 5.13) 22.78
Gun et al., 2004 1.72 (0.96, 2.84) 40.41
Subtotal (I-squared = 0.0%, p = 0.810) 1.81 (1.28, 2.55) 100.00
NOTE: Weights are from random effects analysis
.12
1
8.33

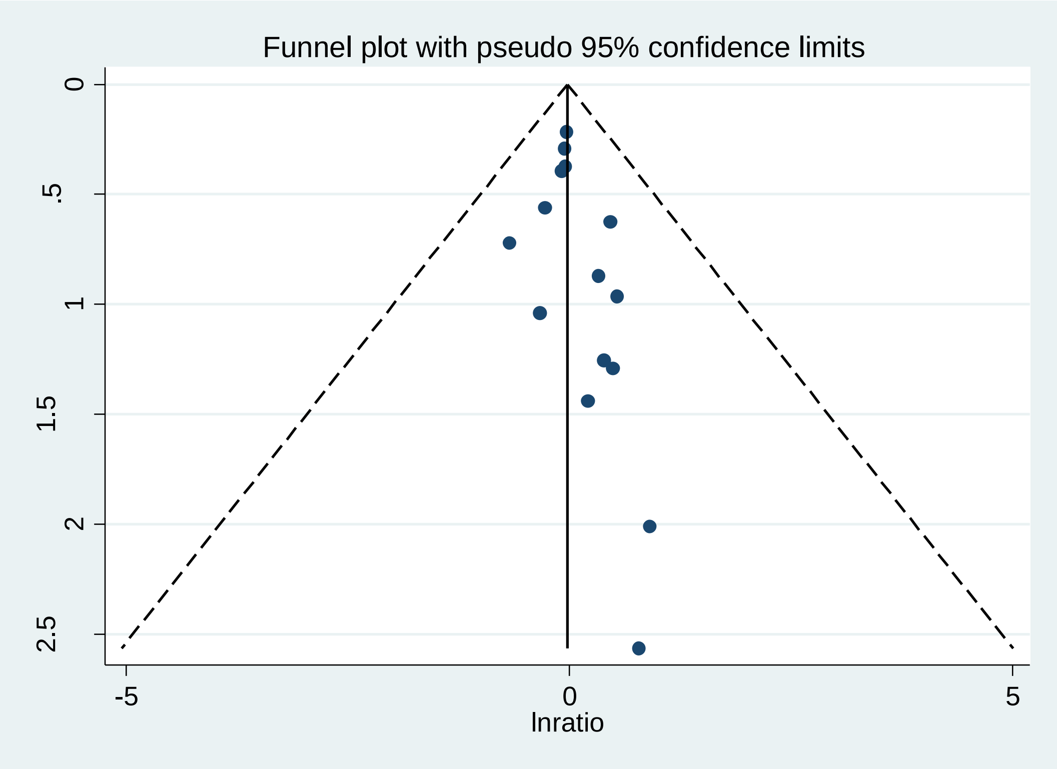

Funnel plot with pseudo 95% confidence limits
0
.5
1
1.5
2
2.5
-5
0
5
lnratio

Figure S1j: Forest and funnel plot of studies for Multiple myeloma incidence and mortality among petroleum workers. Egger's regression test Multiple myeloma incidence and mortality publication bias P = 0.162

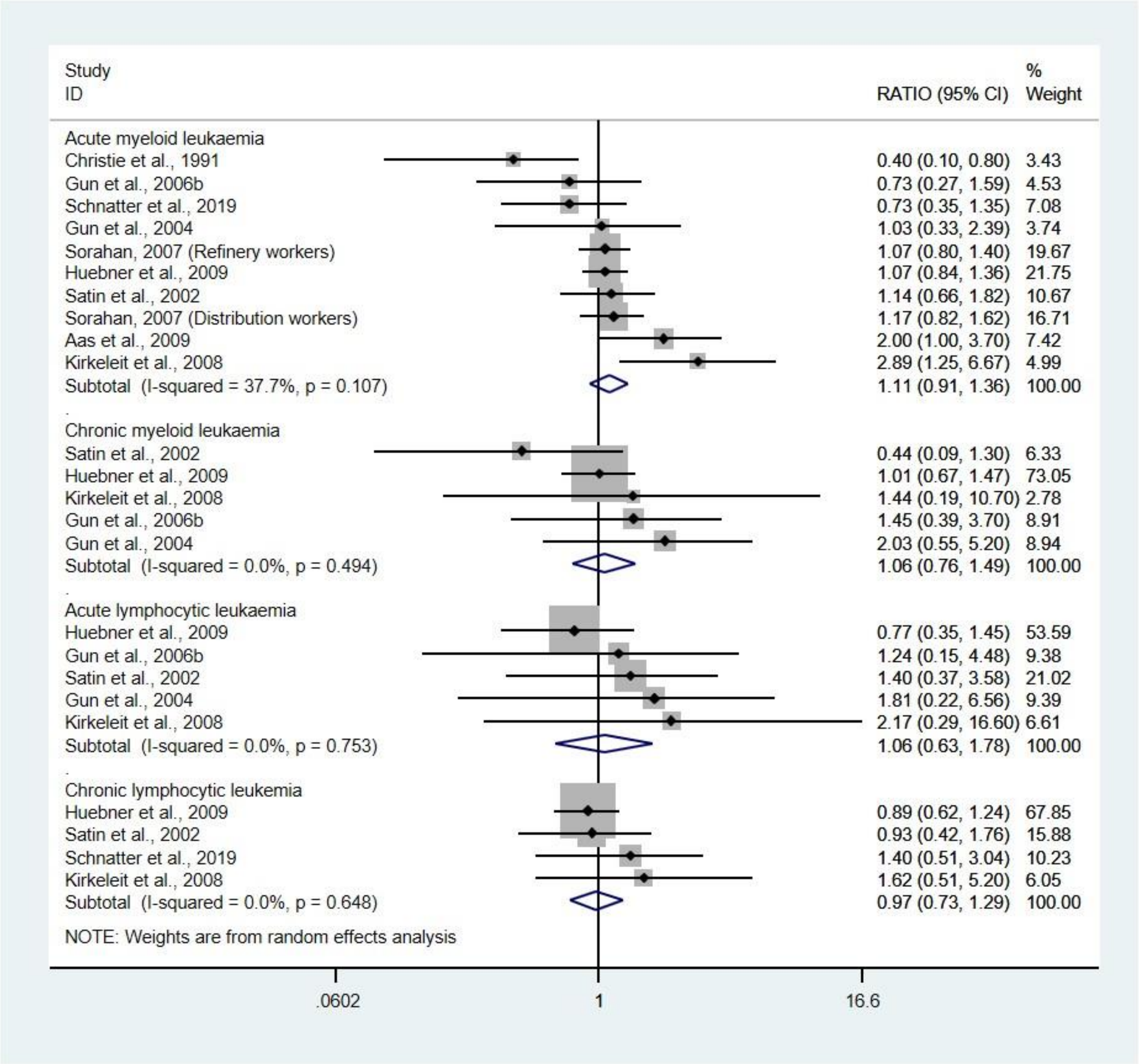

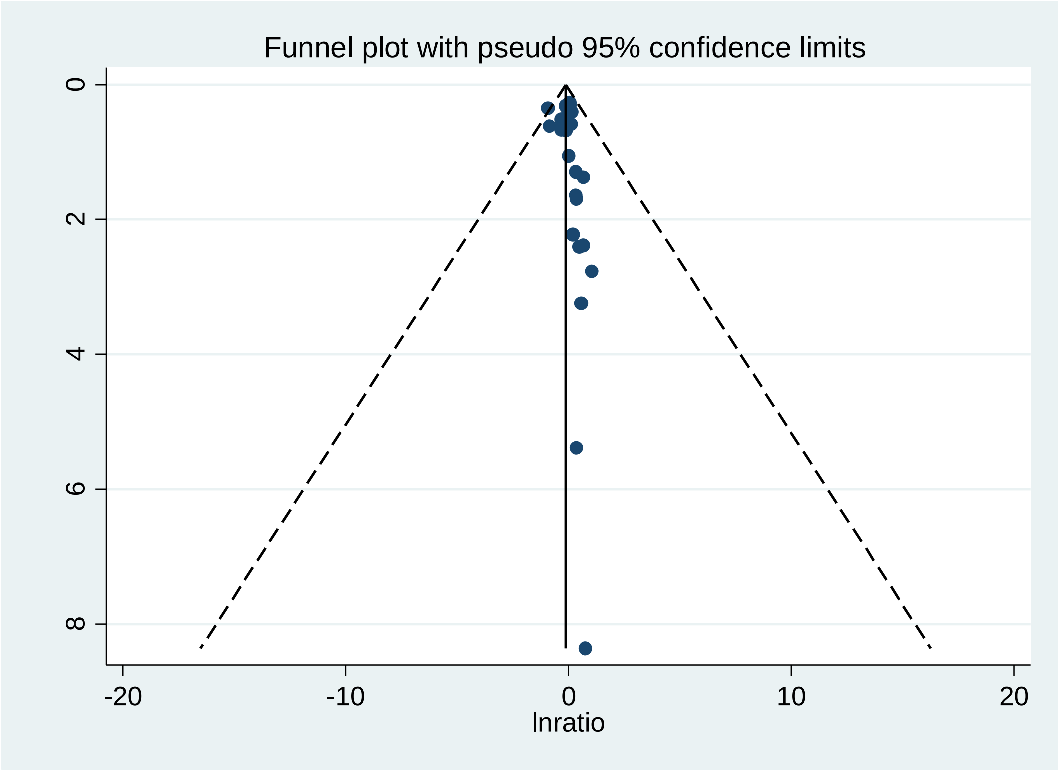

Figure S1k: Forest and funnel plot of studies for subtypes of leukaemia incidence and mortality among petroleum workers Egger's regression test leukaemia subtype incidence and mortality publication bias P = 0.355

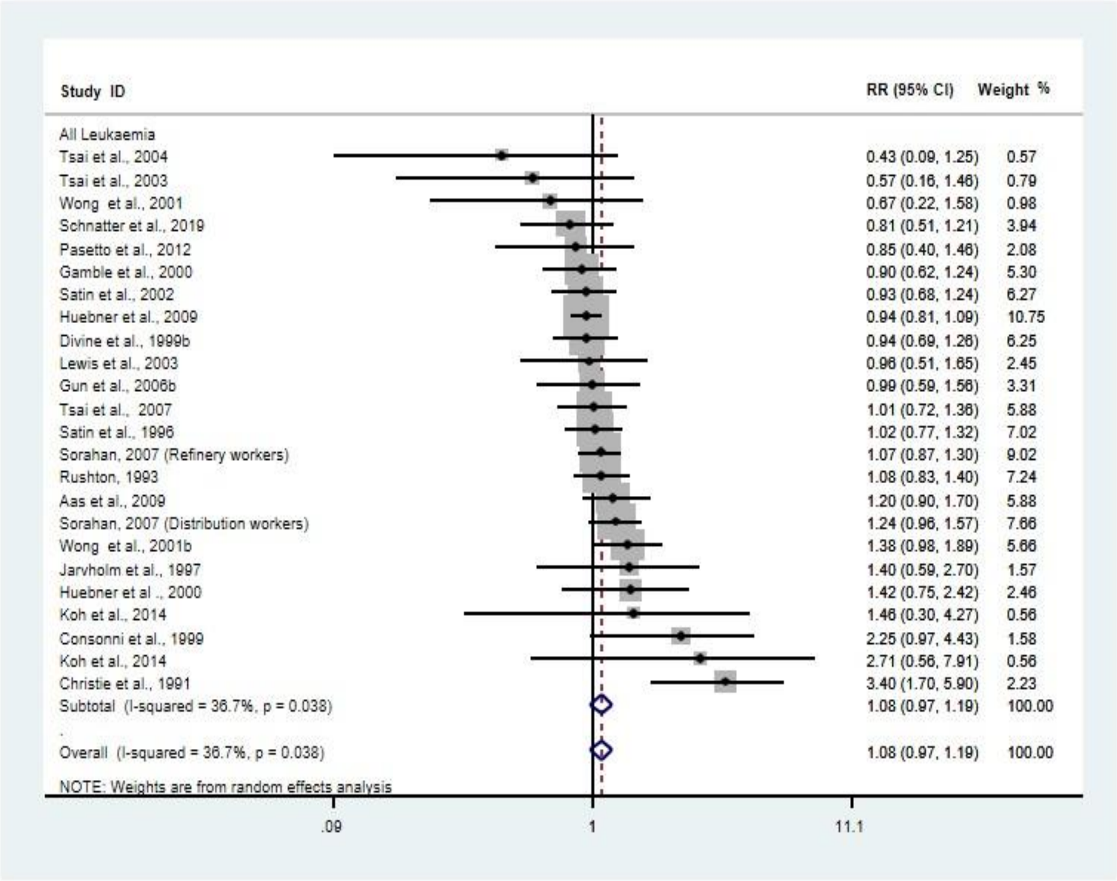

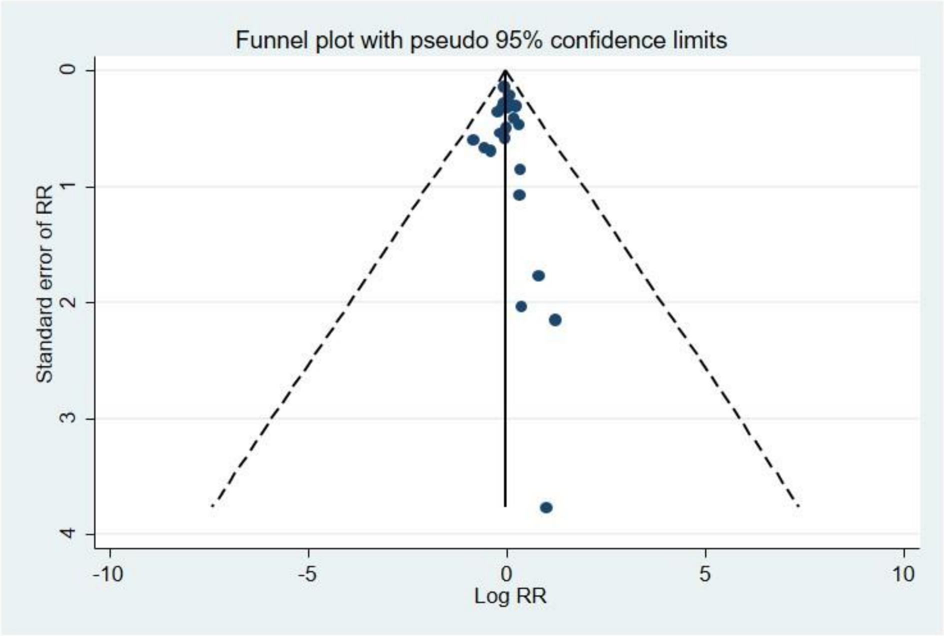

Figure S1l: Forest and funnel plot of studies of leukaemia incidence and mortality among petroleum workers.

Egger's regression test Leukaemia incidence and mortality publication bias P = 0.592

Figure S2: Forest and funnel plot of studies evaluating cancer incidence and mortality among residents of oil producing communities.

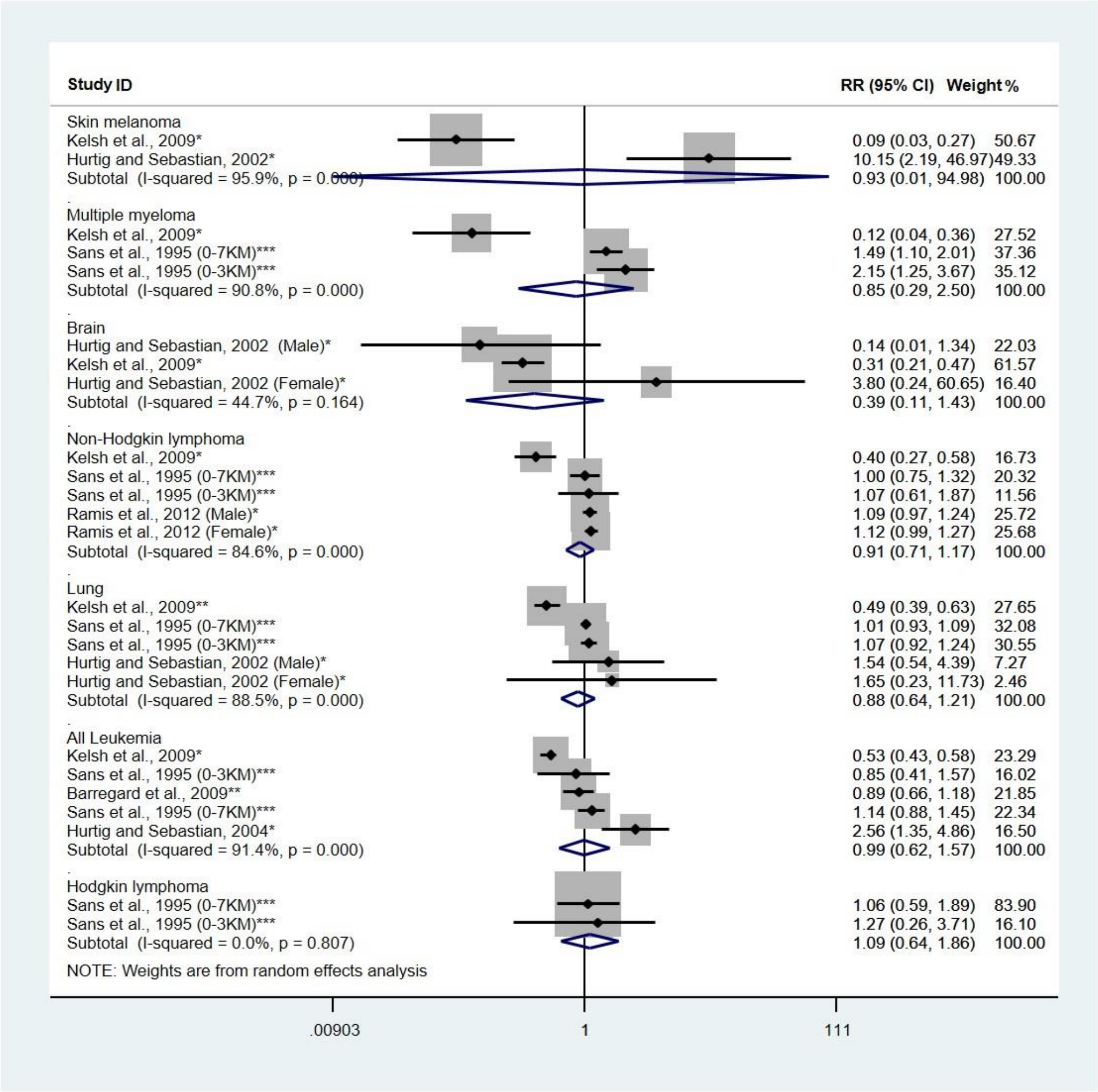

** Relative risk ** incidence studies *** mortality studies*

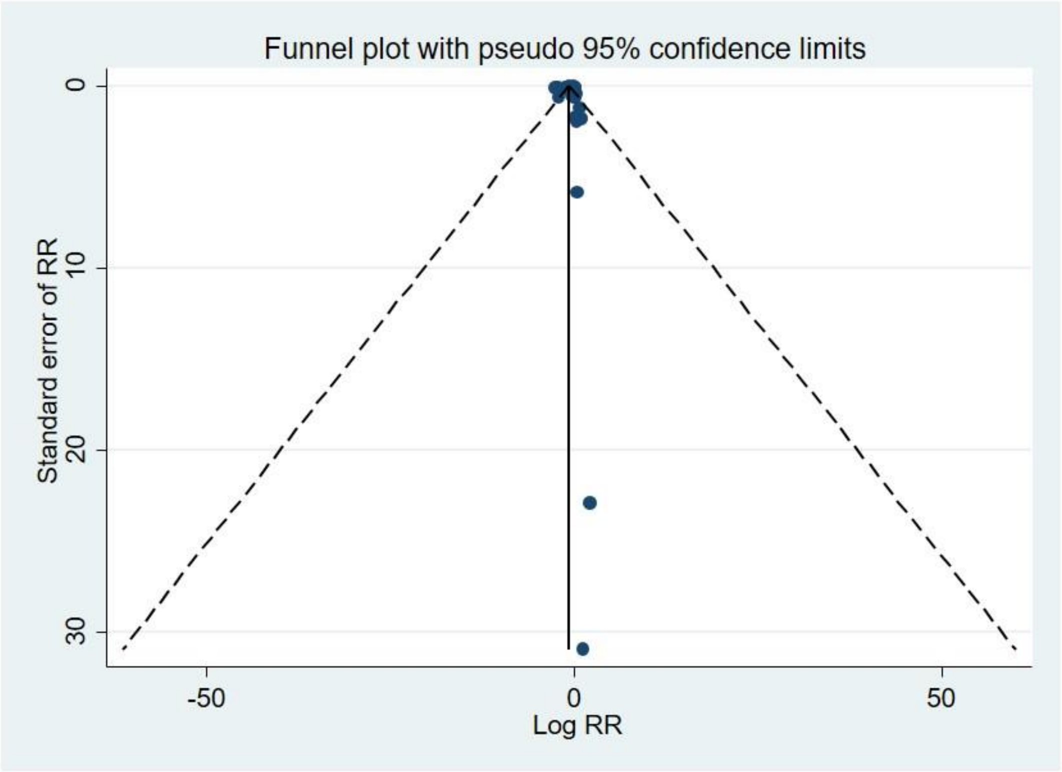

Figure S2: Forest and funnel plot of studies evaluating cancer incidence and mortality among residents of oil producing communities. Egger's regression test for publication bias on all cancers for residents living in oil producing communities P = 0.755

Figure S3: Forest plot of nested case-control studies evaluating cancer among petroleum industry workers and case-control studies evaluating cancer risk among residents living in proximity to petroleum facilities

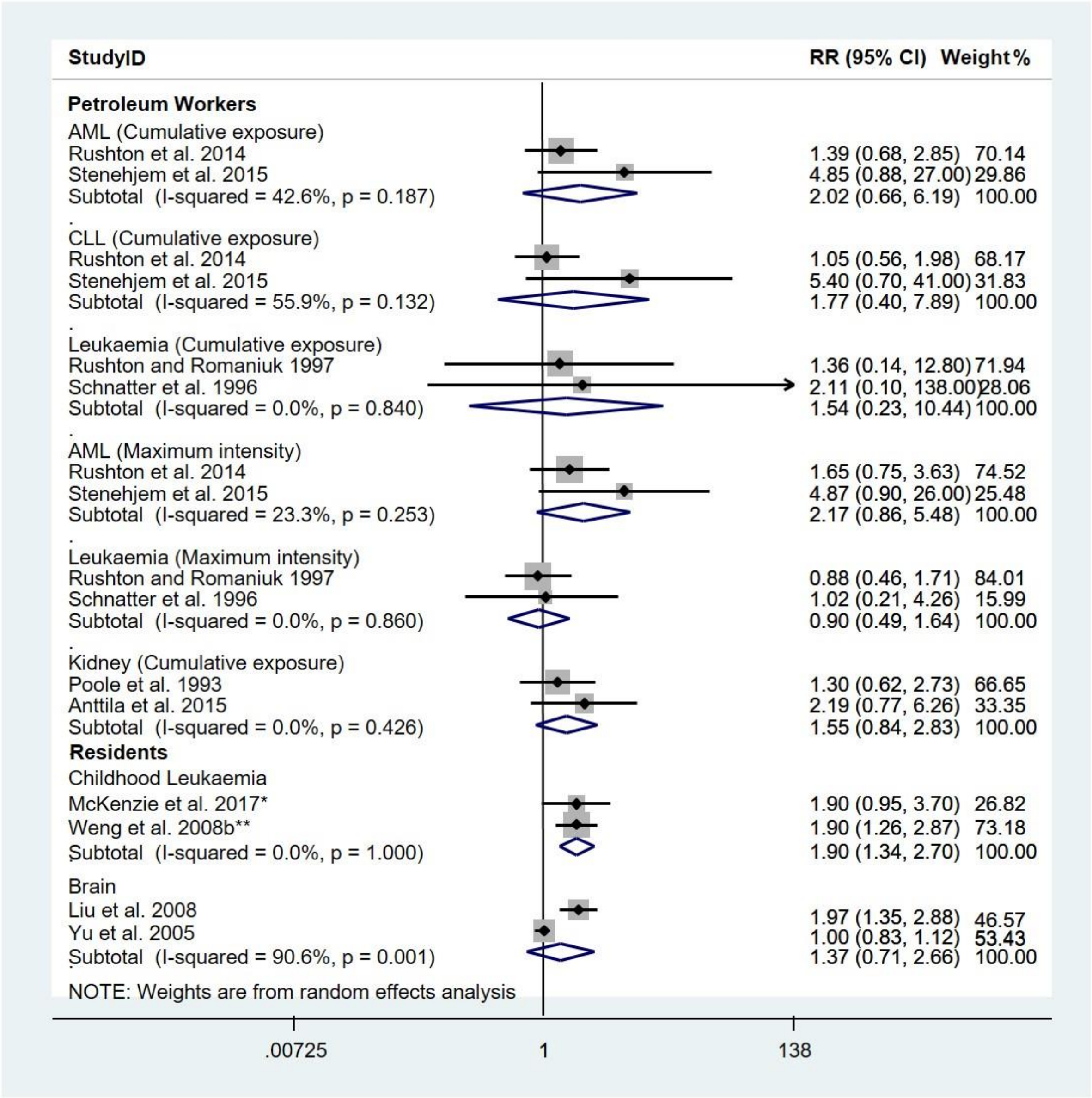

** Childhood leukaemia (Age 0-24); ** Childhood leukaemia (Age 0-19)*

Figure S3: Forest plot of nested case-control studies evaluating cancer among petroleum industry workers and case-control studies evaluating cancer risk among residents living in proximity to petroleum facilities

References

9. Gun, R.; Pratt, N.L.; Roder, D.M.; Ryan, P. Asbestos-related cancers in refinery workers in the Australian petroleum industry. *Archives of environmental & occupational health* **2006**, *61*, 11-16, doi:10.3200/aeoh.61.1.11-16.

18. Christie, D.; Robinson, K.; Gordon, I.; Bisby, J. A prospective study in the Australian petroleum industry. II. Incidence of cancer. *British journal of industrial medicine* **1991**, *48*, 511-514, doi:10.1136/oem.48.8.511.

19. Gun, R.T.; Pratt, N.L.; Griffith, E.C.; Adams, G.G.; Bisby, J.A.; Robinson, K.L. Update of a prospective study of mortality and cancer incidence in the Australian petroleum industry. *Occupational and environmental medicine* **2004**, *61*, 150-156, doi:10.1136/oem.2002.005199.

20. Gun, R.T.; Pratt, N.; Ryan, P.; Roder, D. Update of mortality and cancer incidence in the Australian petroleum industry cohort. *Occupational and environmental medicine* **2006**, *63*, 476-481, doi:10.1136/oem.2005.023796.

21. Schnatter, A.R.; Katz, A.M.; Nicolich, M.J.; Theriault, G. A retrospective mortality study among Canadian petroleum marketing and distribution workers. *Environmental health perspectives* **1993**, *101 Suppl 6*, 85-99, doi:10.1289/ehp.93101s685.

22. Lewis, R.J.; Schnatter, A.R.; Drummond, I.; Murray, N.; Thompson, F.S.; Katz, A.M.; Jorgensen, G.; Nicolich, M.J.; Dahlman, D.; Theriault, G. Mortality and cancer morbidity in a cohort of Canadian petroleum workers. *Occupational and environmental medicine* **2003**, *60*, 918-928, doi:10.1136/oem.60.12.918.

23. Schnatter, A.R.; Nicolich, M.J.; Lewis, R.J.; Thompson, F.L.; Dineen, H.K.; Drummond, I.; Dahlman, D.; Katz, A.M.; Theriault, G. Lung cancer incidence in Canadian petroleum workers. *Occupational and environmental medicine* **2012**, *69*, 877-882, doi:10.1136/oemed-2011-100641.

24. Schnatter, A.R.; Wojcik, N.C.; Jorgensen, G. Mortality Update of a Cohort of Canadian Petroleum Workers. *Journal of occupational and environmental medicine* **2019**, *61*, 225-238, doi:10.1097/JOM.0000000000001523.

25. Gennaro, V.; Ceppi, M.; Boffetta, P.; Fontana, V.; Perrotta, A. Pleural mesothelioma and asbestos exposure among Italian oil refinery workers. *Scandinavian journal of work, environment & health* **1994**, *20*, 213-215, doi:10.5271/sjweh.1406.

26. Consonni, D.; Pesatori, A.C.; Tironi, A.; Bernucci, I.; Zocchetti, C.; Bertazzi, P.A. Mortality study in an Italian oil refinery: extension of the follow-up. *American journal of industrial medicine* **1999**, *35*, 287-294, doi:10.1002/(sici)1097-0274(199903)35:3<287::aid-ajim9>3.0.co;2-f.

27. Pasetto, R.; Zona, A.; Pirastu, R.; Cernigliaro, A.; Dardanoni, G.; Addario, S.P.; Scondotto, S.; Comba, P. Mortality and morbidity study of petrochemical employees in a polluted site. *Environmental Health* **2012**, *11*, 34, doi:10.1186/1476-069X-11-34.

28. Bonzini, M.; Grillo, P.; Consonni, D.; Cacace, R.; Ancona, C.; Forastiere, F.; Cocco, P.L.; Satta, G.; Boldori, L.; Carugno, M.; et al. Cancer risk in oil refinery workers: a pooled mortality study in Italy. *Medicina Del Lavoro* **2019**, *110*, 3-10, doi:10.23749/mdl.v110i1.7842.

29. Koh, D.-H.; Kim, T.-W.; Yoon, Y.-H.; Shin, K.-S.; Yoo, S.-W. Lymphohematopoietic Cancer Mortality and Morbidity of Workers in a Refinery/Petrochemical Complex in Korea. *Safety and health at work* **2011**, *2*, 26-33, doi:https://doi.org/10.5491/SHAW.2011.2.1.26.

30. Koh, D.H.; Chung, E.K.; Jang, J.K.; Lee, H.E.; Ryu, H.W.; Yoo, K.M.; Kim, E.A.; Kim, K.S. Cancer incidence and mortality among temporary maintenance workers in a refinery/petrochemical complex in Korea. *International journal of occupational and environmental health* **2014**, *20*, 141-145, doi:10.1179/2049396714y.0000000059.

31. Aas, G.B.; Aagnes, B.; Strand, L.A.; Grimsrud, T.K. Suggested excess of occupational cancers in Norwegian offshore workers: preliminary results from the Cancer Registry Offshore Cohort. *Scandinavian journal of work, environment & health* **2009**, *35*, 397-399, doi:10.5271/sjweh.1341.

32. Stenehjem, J.S.; Kjaerheim, K.; Rabanal, K.S.; Grimsrud, T.K. Cancer incidence among 41,000 offshore oil industry workers. *Occupational medicine (Oxford, England)* **2014**, *64*, 539-545, doi:10.1093/occmed/kqu111.

33. Kirkeleit, J.; Riise, T.; Bratveit, M.; Moen, B.E. Increased risk of acute myelogenous leukemia and multiple myeloma in a historical cohort of upstream petroleum workers exposed to crude oil. *Cancer causes & control : CCC* **2008**, *19*, 13-23, doi:10.1007/s10552-007-9065-x.

34. Jarvholm, B.; Mellblom, B.; Norrman, R.; Nilsson, R.; Nordlinder, R. Cancer incidence of workers in the Swedish petroleum industry. *Occupational and environmental medicine* **1997**, *54*, 686-691, doi:10.1136/oem.54.9.686.

35. Rushton, L. A 39-YEAR FOLLOW-UP OF THE UK OIL REFINERY AND DISTRIBUTION CENTER STUDIES - RESULTS FOR KIDNEY CANCER AND LEUKEMIA. *Environmental health perspectives* **1993**, *101*, 77-84, doi:10.1289/ehp.93101s677.

36. Sorahan, T. Mortality of UK oil refinery and petroleum distribution workers, 1951-2003. *Occupational medicine (Oxford, England)* **2007**, *57*, 177-185, doi:10.1093/occmed/kql168.

37. Satin, K.P.; Wong, O.; Yuan, L.A.; Bailey, W.J.; Newton, K.L.; Wen, C.P.; Swencicki, R.E. A 50-year mortality follow-up of a large cohort of oil refinery workers in Texas. *Journal of occupational and environmental medicine* **1996**, *38*, 492-506, doi:10.1097/00043764-199605000-00010.

38. Divine, B.J.; Hartman, C.M.; Wendt, J.K. Update of the Texaco Mortality Study 1947-93: Part II. Analyses of Specific Causes of Death for White Men Employed in Refining, Research, and Petrochemicals. *Occupational and environmental medicine* **1999**, *56*, 174-180.

39. Gamble, J.F.; Lewis, R.J.; Jorgensen, G. Mortality among three refinery/petrochemical plant cohorts. II. Retirees. *Journal of occupational and environmental medicine* **2000**, *42*, 730-736, doi:10.1097/00043764-200007000-00008.

40. Huebner, W.W.; Chen, V.W.; Friedlander, B.R.; Wu, X.C.; Jorgensen, G.; Bhojani, F.A.; Friedmann, C.H.; Schmidt, B.A.; Sales, E.A.; Joy, J.A.; et al. Incidence of lymphohaematopoietic malignancies in a petrochemical industry cohort: 1983-94 follow up. *Occupational and environmental medicine* **2000**, *57*, 605-614, doi:10.1136/oem.57.9.605.

41. Sathiakumar, N.; Delzell, E.; Rodu, B.; Beall, C.; Myers, S. Cancer incidence among employees at a petrochemical research facility. *Journal of occupational and environmental medicine* **2001**, *43*, 166-174, doi:10.1097/00043764-200102000-00017.

42. Wong, O.; Harris, F.; Rosamilia, K.; Raabe, G.K. Updated mortality study of workers at a petroleum refinery in Torrance, California, 1959 to 1997. *Journal of occupational and environmental medicine* **2001**, *43*, 1089-1102, doi:10.1097/00043764-200112000-00011.

43. Satin, K.P.; Bailey, W.J.; Newton, K.L.; Ross, A.Y.; Wong, O. Updated epidemiological study of workers at two California petroleum refineries, 1950-95. *Occupational and environmental medicine* **2002**, *59*, 248-256, doi:10.1136/oem.59.4.248.

44. Tsai, S.S.; Yu, H.S.; Liu, C.C.; Yang, C.Y. Increased incidence of preterm delivery in mothers residing in an industrialized area in Taiwan. *Journal of Toxicology and Environmental Health-Part a-Current Issues* **2003**, *66*, 987-994, doi:10.1080/15287390306396.

45. Huebner, W.W.; Wojcik, N.C.; Rosamilia, K.; Jorgensen, G.; Milano, C.A. Mortality updates (1970-1997) of two refinery/petrochemical plant cohorts at Baton Rouge, Louisiana, and Baytown, Texas. *Journal of occupational and environmental medicine* **2004**, *46*, 1229-1245.

46. Buffler, P.A.; Kelsh, M.; Chapman, P.; Wood, S.; Lau, E.; Golembesky, A.; Wood, R.; Kalmes, R.; Brorby, G. Primary brain tumor mortality at a petroleum exploration and extraction research facility. *Journal of occupational and environmental medicine* **2004**, *46*, 257-270, doi:10.1097/01.jom.0000116816.09199.6d.

47. Tsai, S.P.; Chen, V.W.; Fox, E.E.; Wendt, J.K.; Wu, X.C.; Foster, D.E.; Fraser, A.E. Cancer incidence among refinery and petrochemical employees in Louisiana, 1983-1999. *Annals of epidemiology* **2004**, *14*, 722-730, doi:10.1016/j.annepidem.2004.01.005.

48. Tsai, S.P.; Ahmed, F.S.; Wendt, J.K.; Foster, D.E.; Donnelly, R.P.; Strawmyer, T.R. A 56-Year Mortality Follow-Up of Texas Petroleum Refinery and Chemical Employees, 1948–2003. *Journal of occupational and environmental medicine* **2007**, *49*, 557-567, doi:10.1097/JOM.0b013e318057777c.

49. Huebner, W.W.; Wojcik, N.C.; Jorgensen, G.; Marcella, S.P.; Nicolich, M.J. Mortality patterns and trends among 127,266 U.S.-based men in a petroleum company: update 1979-2000. *Journal of occupational and environmental medicine* **2009**, *51*, 1333-1348, doi:10.1097/JOM.0b013e3181be6c18.

50. Divine, B.J.; Hartman, C.M.; Wendt, J.K. Update of the Texaco mortality study 1947-93: Part I. Analysis of overall patterns of mortality among refining, research, and petrochemical workers. *Occupational and environmental medicine* **1999**, *56*, 167, doi:10.1136/oem.56.3.167.

51. Wong, O.; Harris, F.; Rosamilia, K.; Raabe, G.K. An Updated Mortality Study of Workers at a Petroleum Refinery in Beaumont, Texas, 1945 to 1996. *Journal of occupational and environmental medicine* **2001**, *43*, 384-401.

52. Finkelstein, M.M. Asbestos-associated cancers in the Ontario refinery and petrochemical sector. *American journal of industrial medicine* **1996**, *30*, 610-615, doi:10.1002/(sici)1097-0274(199611)30:5<610::Aid-ajim9>3.0.Co;2-w.

53. Schnatter, A.R.; Armstrong, T.W.; Nicolich, M.J.; Thompson, F.S.; Katz, A.M.; Huebner, W.W.; Pearlman, E.D. Lymphohaematopoietic malignancies and quantitative estimates of exposure to benzene in Canadian petroleum distribution workers. *Occupational and environmental medicine* **1996**, *53*, 773-781, doi:10.1136/oem.53.11.773.

54. Anttila, A.; Pokhrel, A.; Heikkila, P.; Viinanen, R.; Pukkala, E. Kidney cancer risk in oil refining in Finland: a nested case-referent study. *Journal of occupational and environmental medicine* **2015**, *57*, 68-72, doi:10.1097/jom.0000000000000301.

55. Rushton, L.; Schnatter, A.R.; Tang, G.; Glass, D.C. Acute myeloid and chronic lymphoid leukaemias and exposure to low-level benzene among petroleum workers. *British journal of cancer* **2014**, *110*, 783-787, doi:10.1038/bjc.2013.780.

56. Glass, D.C.; Schnatter, A.R.; Tang, G.; Irons, R.D.; Rushton, L. Risk of myeloproliferative disease and chronic myeloid leukaemia following exposure to low-level benzene in a nested case-control study of petroleum workers. *Occupational and environmental medicine* **2014**, *71*, 266-274, doi:10.1136/oemed-2013-101664.

57. Stenehjem, J.S.; Kjaerheim, K.; Bratveit, M.; Samuelsen, S.O.; Barone-Adesi, F.; Rothman, N.; Lan, Q.; Grimsrud, T.K. Benzene exposure and risk of lymphohaematopoietic cancers in 25 000 offshore oil industry workers. *British journal of cancer* **2015**, *112*, 1603-1612, doi:10.1038/bjc.2015.108.

58. Rushton, L.; Romaniuk, H. A case-control study to investigate the risk of leukaemia associated with exposure to benzene in petroleum marketing and distribution workers in the United Kingdom. *Occupational and environmental medicine* **1997**, *54*, 152-166, doi:10.1136/oem.54.3.152.

59. Poole, C.; Dreyer, N.A.; Satterfield, M.H.; Levin, L.; Rothman, K.J. Kidney cancer and hydrocarbon exposures among petroleum refinery workers. *Environmental health perspectives* **1993**, *101 Suppl 6*, 53-62, doi:10.1289/ehp.93101s653.

60. Hurtig, A.K.; San Sebastian, M. Geographical differences in cancer incidence in the Amazon basin of Ecuador in relation to residence near oil fields. *International journal of epidemiology* **2002**, *31*, 1021-1027, doi:10.1093/ije/31.5.1021.

61. Hurtig, A.K.; San Sebastian, M. Incidence of childhood leukemia and oil exploitation in the Amazon basin of Ecuador. *International journal of occupational and environmental health* **2004**, *10*, 245-250, doi:10.1179/oeh.2004.10.3.245.

62. Kelsh, M.A.; Morimoto, L.; Lau, E. Cancer mortality and oil production in the Amazon Region of Ecuador, 1990-2005. *International archives of occupational and environmental health* **2009**, *82*, 381-395, doi:10.1007/s00420-008-0345-x.

63. Ramis, R.; Diggle, P.; Boldo, E.; Garcia-Perez, J.; Fernandez-Navarro, P.; Lopez-Abente, G. Analysis of matched geographical areas to study potential links between environmental exposure to oil refineries and non-Hodgkin lymphoma mortality in Spain. *International journal of health geographics* **2012**, *11*, 4, doi:10.1186/1476-072x-11-4.

64. Barregard, L.; Holmberg, E.; Sallsten, G. Leukaemia incidence in people living close to an oil refinery. *Environmental research* **2009**, *109*, 985-990, doi:10.1016/j.envres.2009.09.001.

65. Sans, S.; Elliott, P.; Kleinschmidt, I.; Shaddick, G.; Pattenden, S.; Walls, P.; Grundy, C.; Dolk, H. Cancer incidence and mortality near the Baglan Bay petrochemical works, South Wales. *Occupational and environmental medicine* **1995**, *52*, 217-224, doi:10.1136/oem.52.4.217.

66. Choi, K.H.; Park, M.S.; Ha, M.; Hur, J.I.; Cheong, H.K. Cancer Incidence Trend in the Hebei Spirit Oil Spill Area, from 1999 to 2014: An Ecological Study. *International journal of environmental research and public health* **2018**, *15*, doi:10.3390/ijerph15051006.

67. Lyons, R.A.; Monaghan, S.P.; Heaven, M.; Littlepage, B.N.; Vincent, T.J.; Draper, G.J. Incidence of leukaemia and lymphoma in young people in the vicinity of the petrochemical plant at Baglan Bay, South Wales, 1974 to 1991. *Occupational and environmental medicine* **1995**, *52*, 225-228, doi:10.1136/oem.52.4.225.

68. Tsai, S.S.; Tiao, M.M.; Kuo, H.W.; Wu, T.N.; Yang, C.Y. Association of bladder cancer with residential exposure to petrochemical air pollutant emissions in Taiwan. *Journal of toxicology and environmental health. Part A* **2009**, *72*, 53-59, doi:10.1080/15287390802476934.

69. Weng, H.H.; Tsai, S.S.; Chiu, H.F.; Wu, T.N.; Yang, C.Y. Association of childhood leukemia with residential exposure to petrochemical air pollution in taiwan. *Inhalation toxicology* **2008**, *20*, 31-36, doi:10.1080/08958370701758734.

70. Liu, C.C.; Chen, C.C.; Wu, T.N.; Yang, C.Y. Association of brain cancer with residential exposure to petrochemical air pollution in Taiwan. *Journal of toxicology and environmental health. Part A* **2008**, *71*, 310-314, doi:10.1080/15287390701738491.

71. Yu, C.L.; Wang, S.F.; Pan, P.C.; Wu, M.T.; Ho, C.K.; Smith, T.J.; Li, Y.; Pothier, L.J.; Christiani, D.C. No association between residential exposure to petrochemicals and brain tumor risk. *Cancer epidemiology, biomarkers & prevention : a publication of the American Association for Cancer Research, cosponsored by the American Society of Preventive Oncology* **2005**, *14*, 3007-3009, doi:10.1158/1055-9965.Epi-05-0594.

72. Micheli, A.; Meneghini, E.; Mariottini, M.; Baldini, M.; Baili, P.; Di Salvo, F.; Sant, M. Risk of death for hematological malignancies for residents close to an Italian petrochemical refinery: a population-based case-control study. *Cancer causes & control : CCC* **2014**, *25*, 1635-1644, doi:10.1007/s10552-014-0468-1.

73. McKenzie, L.M.; Allshouse, W.B.; Byers, T.E.; Bedrick, E.J.; Serdar, B.; Adgate, J.L. Childhood hematologic cancer and residential proximity to oil and gas development. *PloS one* **2017**, *12*, e0170423, doi:10.1371/journal.pone.0170423.
